# Adaptive responses of erythritol-producing *Yarrowia lipolytica* to thermal stress after evolution

**DOI:** 10.1007/s00253-024-13103-8

**Published:** 2024-03-15

**Authors:** Kai Xia, Yuqing Chen, Fangmei Liu, Xuequn Zhao, Ruyi Sha, Jun Huang

**Affiliations:** 1 School of Biological and Chemical Engineering, Zhejiang University of Science and Technology, Hangzhou, 310023 China; 2 Key Laboratory of Chemical and Biological Processing Technology for Farm Products of Zhejiang Province, Zhejiang University of Science and Technology, Hangzhou, 310023 China; 3 Zhejiang Provincial Collaborative Innovation Center of Agricultural Biological Resources Biochemical Manufacturing, Zhejiang University of Science and Technology, Hangzhou, 310023 China

**Keywords:** *Yarrowia lipolytica*, Transcriptome, Ceramide, Adaptive laboratory evolution, Branched amino acid

## Abstract

**Abstract:**

Elucidation of the thermotolerance mechanism of erythritol-producing *Yarrowia lipolytica* is of great significance to breed robust industrial strains and reduce cost. This study aimed to breed thermotolerant *Y. lipolytica* and investigate the mechanism underlying the thermotolerant phenotype. *Yarrowia lipolytica* HT34, *Yarrowia lipolytica* HT36, and *Yarrowia lipolytica* HT385 that were capable of growing at 34 °C, 36 °C, and 38.5 °C, respectively, were obtained within 150 days (352 generations) by adaptive laboratory evolution (ALE) integrated with ^60^Co-γ radiation and ultraviolet ray radiation. Comparative genomics analysis showed that genes involved in signal transduction, transcription, and translation regulation were mutated during adaptive evolution. Further, we demonstrated that thermal stress increased the expression of genes related to DNA replication and repair, ceramide and steroid synthesis, and the degradation of branched amino acid (BCAA) and free fatty acid (FFA), while inhibiting the expression of genes involved in glycolysis and the citrate cycle. Erythritol production in thermotolerant strains was remarkably inhibited, which might result from the differential expression of genes involved in erythritol metabolism. Exogenous addition of BCAA and soybean oil promoted the growth of HT385, highlighting the importance of BCAA and FFA in thermal stress response. Additionally, overexpression of 11 out of the 18 upregulated genes individually enabled *Yarrowia lipolytica* CA20 to grow at 34 °C, of which genes *A000121*, *A003183*, and *A005690* had a better effect. Collectively, this study provides novel insights into the adaptation mechanism of *Y. lipolytica* to thermal stress, which will be conducive to the construction of thermotolerant erythritol-producing strains.

**Key points:**

*• ALE combined with mutagenesis is efficient for breeding thermotolerant Y. lipolytica*

*• Genes encoding global regulators are mutated during thermal adaptive evolution*

*• Ceramide and BCAA are critical molecules for cells to tolerate thermal stress*

**Supplementary Information:**

The online version contains supplementary material available at 10.1007/s00253-024-13103-8.

## Introduction

Excessive consumption of sugar has caused serious health problems, such as obesity, diabetes, and metabolic syndrome (Wolnerhanssen et al. 2020). Using alternative sweeteners with low caloric properties to sucrose has thus been an important way to overcome this effect (Liu et al. 2022a). Under this background, sugar alcohols have found their way into our daily life, e.g., mannitol, erythritol, and xylitol, among which erythritol has been gaining more popularity since its consumption does not affect insulin or glucose level and it provides a higher digestive level (Daza-Serna et al. 2021; Erian and Sauer 2022). These have contributed to the rapid growing market of erythritol in recent years; as of 2023, the production value of erythritol is expected to exceed 150 million USD (Liang et al. 2023a). At present, erythritol production on an industrial scale has been well established by fermentation using the non-conventional yeast *Yarrowia lipolytica* (Abbasi et al. 2021; Park and Ledesma-Amaro 2023). Nevertheless, the low productivity, a relatively expensive substrate, and an optimal growth temperature of ~ 30 °C render the production of erythritol by *Y. lipolytica* to be less cost-effective (Ahuja and Rawat 2020; Liang et al. 2023a). Hence, breeding robust strains capable of reducing production cost is of great interest.

Multiple strategies have been adopted to achieve a cost-efficient way for erythritol production, such as using alternative carbon sources (e.g., crop waste and wasting cooking oil) (Liu et al. 2017, 2022b), enhancing erythritol productivity through metabolic engineering (Carly et al. 2017; Mironczuk et al. 2019), and minimizing byproduct formation (Cheng et al. 2018). Besides these, breeding thermotolerant strains is another attractive way. For one thing, fermentation at high temperatures will assist with sterility and the reduction of cooling costs. For another, concerning consolidated bioprocessing, fermentation at high temperatures enables the saccharification and fermentation processes to proceed simultaneously, which will increase efficiencies and reduce cost (Montano Lopez et al. 2022; Yang et al. 2023). Moreover, production of erythritol from lignocellulosic biomass using *Y. lipolytica* is also favored at a higher temperature (Liang et al. 2023b). In the past years, some groups have obtained thermotolerant *Y. lipolytica* by introducing heat-resistant devices, and the growth temperature of *Y. lipolytica* has been elevated from 30 to ~ 35 °C, which is economically valuable (Wang et al. 2020; Zhang et al. 2022; Liang et al. 2023b). However, thermotolerant *Y. lipolytica* capable of growing at a temperature higher than 35 °C is generally hard to obtain by simply expressing or over-expressing exogenous heat-resistant proteins. Adaptive laboratory evolution (ALE) is another promising way to screen phenotype-favorable strains, through which a *Y. lipolytica* strain capable of growing at 37 °C has been obtained (Qiu et al. 2021). Besides this, erythritol-producing *Y. lipolytica* with a growth temperature higher than 37 °C is still lacking. Additionally, the genome changes during thermal adaptive evolution remain elusive.

Elucidation of the thermotolerance mechanism is helpful for the breeding of thermotolerant *Y. lipolytica*. At present, what we have known about the defense mechanism of yeast upon thermal stress predominantly comes from the study of *Saccharomyces cerevisiae* (Caspeta et al. 2014; Gao et al. 2016; Muhlhofer et al. 2019; Wang et al. 2022). For the erythritol-producing *Y. lipolytica*, the central carbon metabolism and amino acid metabolism were found to be involved in the thermal stress response at a transcriptional level, while ATP, thiamine, and trehalose were critical molecules for cells to grow under conditions of high temperature (Qiu et al. 2021; Celinska 2022). Beyond these, our understanding of the thermotolerance mechanism of *Y. lipolytica* is still inadequate. In this study, we aimed to breed a stable and thermotolerant *Y. lipolytica* that could grow normally at temperatures close to 40 °C within a relatively short period of time. Further, the genetic changes and transcriptome response of *Y. lipolytica* during thermal adaptation were investigated. Additionally, upregulated genes of interest identified by transcriptome analysis were verified by reverse engineering to clarify if these genes could be directly used for strain engineering.

## Materials and methods

### Bacterial strains, plasmids, and growth conditions

Detailed information of the strains and plasmids used in this study is presented in the Supplementary material (Table S1). *Yarrowia lipolytica* CA20 (deposited in the China Center for Type Culture Collection, No. M2022880) (Liu et al. 2023), *Yarrowia lipolytica* CA20∆*ura3*, *Yarrowia lipolytica* HT34, *Yarrowia lipolytica* HT36, and *Yarrowia lipolytica* HT385 were grown in YPDN medium (10 g/L yeast extract, 5 g/L peptone, 200 g/L D-glucose, 5 g/L NaCl, 0.5 g/L KH_2_PO_4_) at 30, 30, 34, 36, and 38.5 °C, respectively, with shaking at 200 r/min. *Y. lipolytica* CA20∆*ura3* containing pDCXRA, pDCXRA121, pDCXRA184, pDCXRA800, pDCXRA1678, pDCXRA2175, pDCXRA2375, pDCXRA2808, pDCXRA3183, pDCXRA3902, pDCXRA4055, pDCXRA4467, pDCXRA4535, pDCXRA4625, pDCXRA4733, pDCXRA5690, pDCXRA5844, pDCXRA6220, or pDCXRA6279 was grown at YNBD medium (6.7 g/L yeast nitrogen base without amino acids (Sangon Biotech Co., Ltd., Shanghai, China), 0.67 g/L amino acid supplements without uracil (Sigma-Aldrich, MO, USA), and 5 g/L (NH_4_)_2_SO_4_, 20 g/L D-glucose). All *Escherichia coli* strains were grown at 37 °C in LB medium containing 5 g/L yeast extract, 10 g/L peptone, and 10 g/L NaCl, with shaking at 200 r/min. The LB medium was supplemented with ampicillin (50 μg/mL) or chloramphenicol (50 μg/mL) to maintain plasmids where necessary. The activation and growth of all strains were performed in 250-mL Erlenmeyer flasks, unless otherwise indicated. All antibiotics (USP grade) and other chemical reagents (AR grade) were purchased from Sangon Biotech Co., Ltd. (Songjiang, Shanghai, China) and Sinopharm Chemical Reagent Co., Ltd. (Huangpu, Shanghai, China).

### Thermal adaptation evolution

**Fig. 1:**
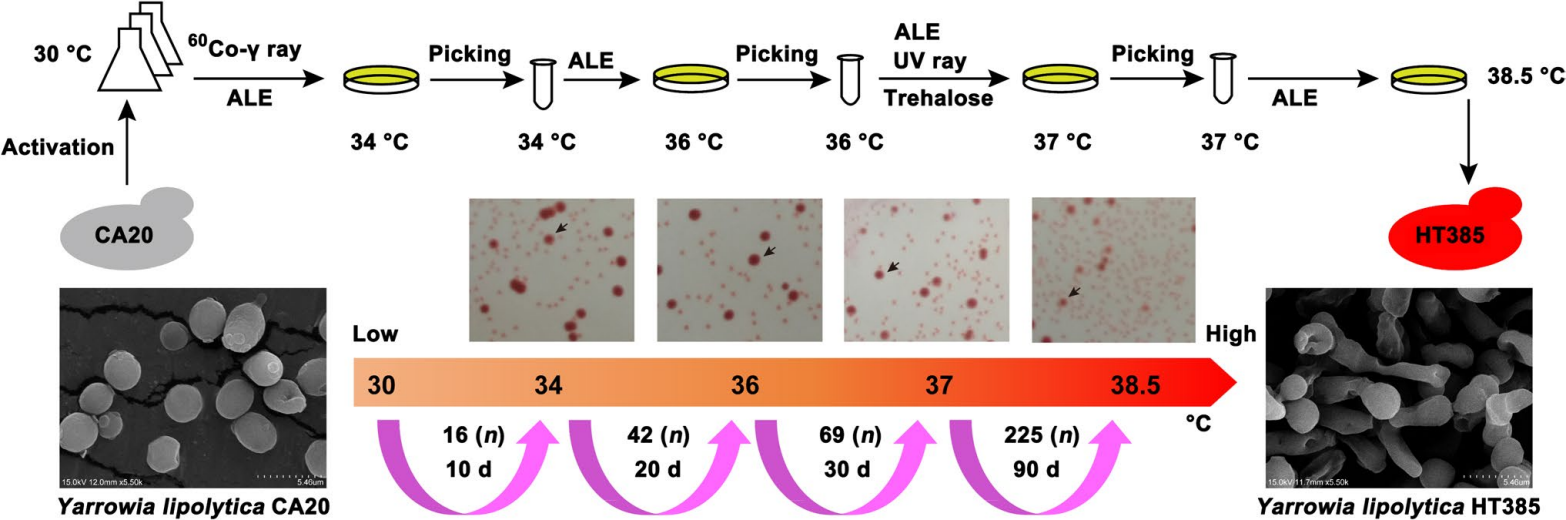
Breeding of thermotolerant *Y. lipolytica* strains by adaptive laboratory evolution (ALE) and mutagenesis

Mutagenesis combined with ALE was adopted for the selection of thermotolerant strains using *Y. lipolytica* CA20 as the parental strain. An outline of this process is shown in Fig. 1. The ALE process was performed as previously described with some modifications (Satomura et al. 2013). The mutagenesis using ^60^Co-γ radiation and ultraviolet (UV) ray radiation was carried out according to our previous study (Liu et al. 2023). Briefly, the -80 °C stored strain was activated using YPDN medium at 30 °C with shaking at 200 r/min for 36 h. Afterwards, the culture was serially diluted and spread onto the YPDN plate to form single colonies. The large colony was picked out and inoculated into 40-mL YPDN medium, followed by culturing at 30 °C until the cell density (OD_600_) reached 3.0 ± 0.5. Thereafter, the cells were collected by centrifugation (5000 r/min, 3 min) at room temperature. The cells were then suspended using sterilized ddH_2_O and washed twice. Thereafter, the cells were diluted by ddH_2_O to produce a solution with a cell number of ~ 10^7^/mL, followed by treatment using ^60^Co-γ radiation at a dose of 1200 Gy. After treatment, the solution was diluted and spread onto the YPDN plate containing 0.005% (w/v) 2, 3, 5-triphenyl tetrazolium chloride (TTC), followed by incubation at 34 °C for 3–4 days. The largest colony was picked out and inoculated into 40-mL YPDN medium. After culturing at 34 °C for 2 days, the culture was diluted by 1:100 to 40-mL fresh medium and grown at 34 °C for 72 h, during which the growth curve was obtained and compared with that of the *Y. lipolytica* CA20 grown at 30 °C. If the growth of the selected strain under 34 °C was not significantly different from that of CA20, this strain (named as HT34) would be used as the start strain for the next round of adaptive evolution to obtain a strain capable of growing at 36 °C. Otherwise, the strain would be cultured at 34 °C repeatedly until it got adaptation.

For the screening of strains tolerant to 36 °C, a 48-h culture of HT34 was diluted and spread onto the YPDN plate. After incubating at 36 °C for 4–5 days, the largest colony was picked out and cultured as described above. The culture process would be repeated until the growth of the selected strain under 36 °C (named as HT36) was not significantly different from that of HT34 grown at 34 °C. The strain HT36 was used for the selection of strains tolerant to 37 °C, during which UV ray radiation was performed. A 36-h culture of HT36 was diluted and spread onto YPDN plates containing 1 g/L trehalose. The plates were then treated with UV ray (20 w) for 1 min, and the vertical distance between the UV light and plates was 20 cm. Thereafter, the plates were incubated at 37 °C for 4–5 days. Then, the growth curve of strain capable of growing at 37 °C was obtained and compared with that of the HT36. The strain adapted to 37 °C was used for the breeding of strains tolerant to a higher temperature. Finally, the strain adapted to a temperature of 38.5 °C was named as *Y. lipolytica* HT385. To test the stability of thermotolerant phenotype, the strains HT34, HT36, and HT385 were grown at 30 °C for 30 days, during which cultures were transferred every 72 h. Afterwards, the strains were returned to grow at 34, 36, and 38.5 °C, respectively, producing the strains *Yarrowia lipolytica* HT34R, *Yarrowia lipolytica* HT36R, and *Yarrowia lipolytica* HT385R.

The number of generations (*n*) during each growth period was calculated as previously described (Daskalaki et al. 2019), which was approximated based on the cell density (cells/mL) at the beginning (*N*_0_) and at the end (*N*_*t*_) of the period: *N*_*t*_ = *N*_0_ × 2^*n*^.

### Assay of cell growth and morphology

The growth of *Y. lipolytica* CA20, *Y. lipolytica* HT34, *Y. lipolytica* HT36, and *Y. lipolytica* HT385 at different temperatures was determined by monitoring the optical density (OD_600_) using a microtiter plate reader (SpectraMax iD3, Molecular Devices, CA, USA). In brief, a 48-h culture of each strain was diluted by 40 mL of fresh YPDN medium to produce an initial OD_600_ of 0.1 ± 0.02, then the cultures were incubated at designated temperatures for 96 h, during which the OD_600_ was detected every 24 h.

To investigate the influence of glycerol, amino acid, soybean oil, and trehalose on the growth of *Y. lipolytica* HT385, a 48-h culture of HT385 was diluted by 40-mL YPDN medium containing either glycerol (with a final concentration of 5, 10, and 20 g/L), glutamate (1, 10, and 20 μM), leucine (1, 10, and 20 μM), isoleucine (1, 10, and 20 μM), valine (1, 10, and 20 μM), soybean oil (5, 10, and 20 g/L), or trehalose (0.5, 1, and 2 g/L), with an initial OD_600_ of 0.1 ± 0. 02. The OD_600_ of the culture was detected every 24 h. To investigate the effect of ATP and thiamine on the growth of HT385, ATP with a final concentration of 20 mg/L or thiamine of 10 mg/L was added to the medium every 12 h, during which the OD_600_ was monitored.

The growth of *Y. lipolytica* CA20∆*ura3* with an overexpression of the target gene was carried out using YNBD medium. A 48-h culture of each strain grown at 30 °C was diluted by 40 mL of fresh YNBD medium with an initial OD_600_ of 0.1 ± 0.02, followed by incubation at 30, 34, 36, and 38.5 °C for 72 h, respectively, during which the OD_600_ was detected every 12 h.

The cell morphology was observed using a scanning electron microscope (SEM) (Hitachi TM3000, Tokyo, Japan) as previously described with some modifications (Kong et al. 2021). Briefly, a 60-h culture of *Y. lipolytica* CA20 and *Y. lipolytica* HT385 was collected, and the cells were obtained by centrifugation (6000 r/min, 3 min) at room temperature and washed twice using 1 × PBS solution (pH 7.4). Thereafter, the cells were fixed overnight in a PBS solution containing 2.5% (v/v) glutaraldehyde and 2% (v/v) formaldehyde at 4 °C. Afterwards, the cells were washed twice using 1 × PBS, and then dehydrated in solutions with increasing concentrations of ethanol (35, 50, and 75% for 30 min each and two cycles of 90 and 100% for 30 min each). Then, the cells were lyophilized, sputter coated with gold, and observed using the microscope.

### Quantification of superoxide dismutase and catalase activity, ATP, free fatty acid, and amino acid

The intracellular superoxide dismutase (SOD) activity, catalase (CAT) activity, free fatty acid (FFA) content, and amino acid (AA) content were assayed by the SOD activity assay kit (AKAO001M), CAT activity assay kit (AKAO003-1 M), FFA content assay kit (AKFA008M), and AA content assay kit (AKAM001M) that were bought from the Beijing Boxbio Science & Technology Co., Ltd. (Tongzhou, Beijing, China). The detailed operation steps referred to the manufacturer’s instructions. For the sample preparation, a 48-h culture of *Y. lipolytica* CA20 and *Y. lipolytica* HT385 was diluted by 40 mL of fresh YPDN medium, followed by incubation at 30 °C and 38.5 °C for 72 h, respectively. The cells were collected by centrifugation (5000 r/min, 5 min) at room temperature, with an interval of 24 h. Thereafter, the cells were washed twice using distilled water and re-suspended using the extraction buffer provided by the kit. The cells were further lysed by ultrasonication and centrifuged at 8000 × *g* for 10 min at 4 °C. The supernatant was collected and used for detection. The viable cells in each sample were determined by counting colony-forming units (CFU/mL). The results were shown as U/cell (SOD and CAT activity), μM/cell (FFA), and μg/L/cell (AA). The intracellular ATP concentration was detected by the ATP detection kit (S0026) purchased from Shanghai Beyotime Biotechnology Co., Ltd. (Songjiang, Shanghai, China), which was described elsewhere (Qiu et al. 2021). The fluorescence was detected by a microtiter plate reader (SpectraMax iD3, Molecular Devices, CA, USA).

### Erythritol fermentation and detection

The erythritol fermentation was performed as previously described (Liu et al. 2023). Briefly, a 48-h culture of the strains CA20, HT36, and HT385 was diluted by 1:20 into 40 mL of YPDN medium contained in 500-mL Erlenmeyer flasks, respectively, followed by incubation for 120 h with shaking at 200 r/min. Afterwards, the culture was collected, and the supernatant was obtained by centrifugation (6000 r/min, 5 min). Similarly, the erythritol fermentation carried out by *Y. lipolytica* CA20∆*ura3* with an overexpression of the target gene was performed using YNBD medium containing 200 g/L glucose. The concentration of glucose and erythritol was detected by high-performance liquid chromatography (HPLC) (e2695, Waters, Milford, MA) using an ion exclusion column Aminex HPX-87H (Bio-Rad, CA, USA) and a differential refractive index detector (2414, Waters, Milford, MA), with a mobile phase of 0.5 mM H_2_SO_4_. The flow rate and temperature were set at 0.6 mL/min and 35 °C, respectively, and samples with a volume of 10 μL were detected each time.

### Genome sequencing and comparative genomics analysis

In our previous study, the whole genome of *Y. lipolytica* CA20 was sequenced and submitted to the NCBI public database with an accession number of PRJNA957569 (Xia et al. 2023). The genomes of *Y. lipolytica* HT34, *Y. lipolytica* HT36, and *Y. lipolytica* HT385 were re-sequenced and analyzed in this study. Briefly, cells collected from a 48-h culture were used for DNA extraction using the Ezup Column Yeast Genomic DNA Purification Kit (Sangon Biotech, Shanghai, China). The qualified DNA (> 3 µg, concentration > 30 ng/µL, OD_260_/OD_280_ = 1.80 ~ 2.00) was used for the construction of sequencing libraries. Paired-end libraries with inserted sizes of ~ 450 bp were prepared following Illumina’s standard genomic DNA library preparation procedure. After quantification, paired-end libraries were sequenced by Shanghai Biozeron Biothchology Co., Ltd. (Jiading, Shanghai, China) using Illumina HiSeq platforms. The raw paired-end reads were trimmed and quality controlled by Trimmomatic (version 0.39, http://www.usadellab.org/cms/uploads/supplementary/Trimmomatic) with default parameters. The high-quality reads of HT34, HT36, and HT385 were aligned to the CA20 genome sequence using BWA software (version 0.7.12-r1039, http://bio-bwa.sourceforge.net/). The PCR-duplication reads were removed by SAMtools (version 1.4, http://samtools.sourceforge.net/). Thereafter, GATK (version 3.8, HaplotypeCaller, http://www.broadinstitute.org/gatk/) was used to identify single-nucleotide polymorphisms (SNP) and insertions and deletions (Indel). Structure variations (SV) were identified by BreakDancer (version 1.1.2, http://breakdancer.sourceforge.net). The software ANNOVAR (http://www.openbioinformatics.org/annovar/) was used to annotate the detected variations.

### RNA extraction and transcriptome analysis

The transcriptome analysis was performed as described in our previous studies (Xia et al. 2020, 2021). For the sample preparation, a 48-h culture of *Y*. *lipolytica* CA20, *Y*. *lipolytica* HT36, and *Y*. *lipolytica* HT385 was diluted by 1:100 into 40-mL fresh YPDN medium, followed by incubation at 30, 36, and 38.5 °C, respectively, for 72 h. Afterwards, cell samples harvested from three independent cultures were used for the transcriptomic analysis (the samples were named YLX3, YLX6, and YLX8), which was performed by Biozeron Biotechnology Co., Ltd. (Jiading, Shanghai, China). The paired-end libraries were sequenced using the Illumina HiSeq 2000 platforms.

The raw paired-end reads were trimmed and quality controlled by Trimmomatic with parameters (SLIDINGWINDOW: 4: 15 MINLEN: 75) (version 0.36). Then, the clean reads were separately aligned to the genome of *Y*. *lipolytica* CA20 using hisat2 software (https://ccb.jhu.edu/software/hisat2/index.shtml), with default parameters. The data quality was assessed by qualimap (version 2.2.1, http://qualimap.bioinfo.cipf.es/), while the gene reads were counted by htseq (https://htseq.readthedocs.io/en/release_0.11.1/). edgeR was used for the differential expression analysis (Robinson et al. 2010). The differentially expressed genes (DEGs) were selected using the following criteria: |Log_2_FC|≥ 1, the FC (fold change) was calculated by dividing the expression level (FPKM, fragments per kilobase per million mapped fragments) of each gene in YLX8 or YLX6 by that in YLX3, while the false discovery rate (FDR) should be less than 0.05. To understand the function of DEGs, GO functional enrichment and KEGG pathway analysis were carried out by Goatools (Klopfenstein et al. 2018) and KOBAS (Xie et al. 2011), respectively. DEGs were significantly enriched in GO terms and metabolic pathways when their Bonferroni-corrected *p* value was less than 0.05.

### Gene deletion and overexpression

The genome DNA of *Y. lipolytica* CA20 was used as the template for amplifying the desired DNA fragments. The restricted enzymes, PCR systems, and DNA purification kits were purchased from Takara Biomedical Technology (Beijing) Co., Ltd. (Changping, Beijing, China) and Sangon Biotech Co., Ltd. (Songjiang, Shanghai, China). DNA sequencing was performed by Sangon Biotech Co., Ltd. (Songjiang, Shanghai, China). All primers used in this study were listed in Supplementary material (Table S2). Competent cells of *E. coli* DH5α were prepared as described by the Competent Cell Preparation Kit (catalog no. 9139, TaKaRa, Beijing, China). The preparation of *Y. lipolytica* competent cells was carried out as described in the Frozen-EZ Yeast Transformation II™ Kit (catalog no. T2001, Zymo Research, CA, USA).

Deletion of *ura3* was completed by double-crossover homologous recombination using plasmid pHUD (Supplemental Fig. S1). Briefly, a 1.0-μg linearized pHUD was thoroughly mixed with 50 μL competent cells of *Y. lipolytica* CA20 and 500 μL solution 3 provided by the Frozen-EZ Yeast Transformation II™ Kit, followed by incubation at 30 °C with shaking at 400 r/min for 2 h. Thereafter, the mix was spread onto the YPDN plate containing 1 g/L 5-fluoroorotic acid (5-FOA), followed by incubation at 30 °C for 4–5 days. The grown colonies were picked out and cultured for verification by sequencing.

To overexpress target genes of interest in *Y. lipolytica* CA20∆*ura3*, we first constructed the expression vector pDCXRA. The detailed process is shown in the Supplementary material (Fig. S2). Briefly, the yeast expression vector pCRISPRyl purchased from Addgene (catalog no. 70007) was used as a start vector. The pDCXRA was constructed by deletion of the gene coding for Cas9, insertion of the *ura3* expression cassette, and insertion of the autonomously replicating sequence (ARS1). The 18 genes of interest were inserted into the pDCXRA, respectively, using Gibson Assembly Master Mix (New England Biolabs, MA, USA) under the promoter of UAS1B8-TEF. Detailed information of the plasmids and strains was presented in the Supplemental Table S1.

### Statistical analysis

All experiments were conducted with three biological replicates. Statistical analyses were performed using the Origin software (version 9.0) (OriginLab, Northampton, MA, USA). Where appropriate, the data were analyzed using the Student’s *t*-test or analysis of variance (ANOVA) with a Bonferroni’s multiple-comparison test (Xia et al. 2021). Differences were considered statistically significant at *p* < 0.05.

## Results

### Breeding of thermotolerant Y. lipolytica by ALE and mutagenesis

To obtain strains that were able to grow at high temperatures, ALE and mutagenesis were adopted using erythritol-high-producing *Y. lipolytica* CA20 as a start (Liu et al. 2023). The breeding process was divided into four stages, with temperatures ranging from 30 to 34 °C, 34 to 36 °C, 36 to 37 °C, and 37 to 38.5 °C, respectively (Fig. 1). In each stage, diverse methods were applied. To avoid the loss of erythritol synthesis by thermotolerant strains, glucose of 200 g/L was added to the medium to maintain the osmotic stress, and TTC was also added as an indicator (Qiu et al. 2021; Liu et al. 2023). The results showed that it took 150 days (corresponding to 352 generations) to obtain a strain capable of growing at 38.5 °C, during which the time needed for each stage was 10 (16 generations), 20 (42 generations), 30 (69 generations), and 90 (225 generations) days, respectively. The cell shape of CA20 grown at 30 °C was oval, while the shape of HT385 was elongated and oblate when grown at 38.5 °C, indicating a physiological alteration of the cell during adaptive evolution.

**Fig. 2:**
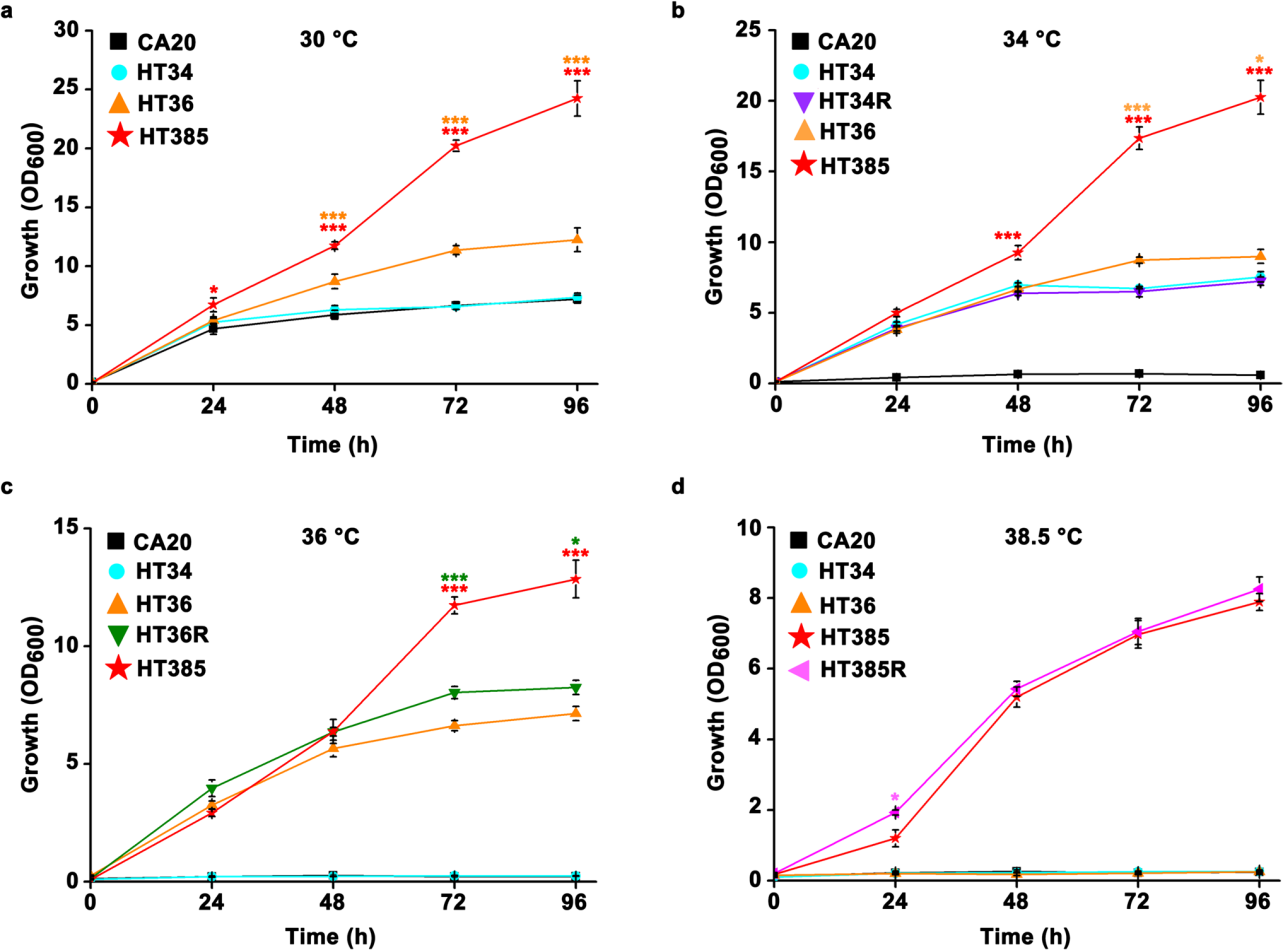
Growth curves of *Y. lipolytica* CA20, *Y. lipolytica* HT34, *Y. lipolytica* HT36, and *Y. lipolytica* HT385 under 30 (**a**), 34 (**b**), 36 (**c**), and 38.5 °C (**d**). HT34, HT36, and HT385 were grown at 30 °C for 30 days, after which the strains were returned to grow at 34, 36, and 38.5 °C, respectively, to produce the strains HT34R, HT36R, and HT385R. Data obtained from three biological replicates were shown as the mean ± standard deviation. Different colors of “*” indicating significant differences are in accordance with the respective strain (*p < 0.05; ***p < 0.001). The control group in **a**, **b**, **c**, and **d** was set as CA20, HT34, HT36, and HT385, respectively

To determine whether the obtained thermotolerant strains were stable or just a tentative phenotype change, we next detected the growth of *Y. lipolytica* HT34, *Y. lipolytica* HT36, and *Y. lipolytica* HT385 under different temperatures. As shown in Fig. 2, the growth of HT34 at 30 °C was similar to that observed for CA20, whereas the strains HT36 and HT385 grew better than HT34 and CA20, especially for HT385 where the maximum OD_600_ was close to 25 after growing for 96 h (Fig. 2a). CA20 was not able to grow at 34 °C, while the strains HT34, HT36, and HT385 grew well (Fig. 2b). The thermotolerant phenotype of HT34 was stable, as there was no significant difference between the growth of HT34 and HT34R. Similarly, the strain HT36 was adapted to 36 °C, as the growth of HT36R was even better than that of HT36 (Fig. 2c). Moreover, the strain HT385 was able to grow at 38.5 °C, regardless of the fact that a longer lag phase was observed (Fig. 2d). There was no significant difference between the growth of HT385R and HT385, implying the adaptation of HT385 to 38.5 °C. Collectively, these results suggest that ALE combined with mutagenesis is an efficient way to breed thermotolerant *Y. lipolytica*.

### Genome changes of Y. lipolytica during thermal adaptive evolution

**Fig. 3:**
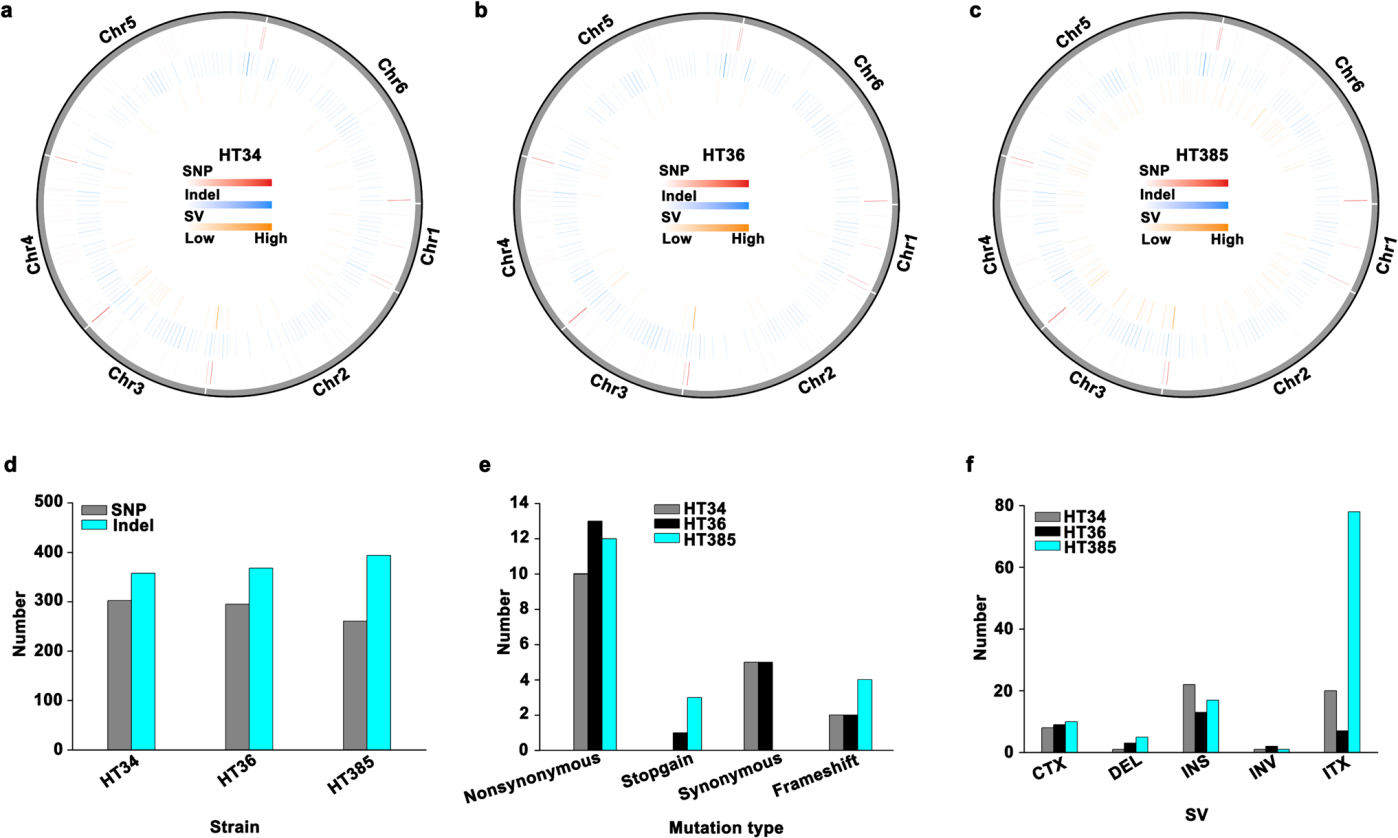
Genome changes of thermotolerant strains during adaptive evolution. An overview map of the SNP, Indel, and SV sites on the chromosomes of HT34 (**a**), HT36 (**b**), and HT385 (**c**). The number of SNP and Indel (**d**), mutated genes (**e**), and SV (**f**). SNP, single-nucleotide polymorphisms; Indel, insertions and deletions; SV, structure variations; CTX, inter-chromosomal translocation; DEL, deletion; INS, insertion; INV, inversion; ITX, intra-chromosomal translocation

After obtaining the thermotolerant *Y. lipolytica*, we next sought to figure out how cells respond to thermal stress at a genomic level. Hence, the genomes of *Y. lipolytica* HT34, *Y. lipolytica* HT36, and *Y. lipolytica* HT385 were sequenced and compared with *Y. lipolytica* CA20 to find genome variants. The results showed that SNP, Indel, and SV were both found in the thermotolerant strains (Fig. 3a–c). The number of SNP and Indel in HT34, HT36, and HT385 was 302/358, 295/368, and 261/394, respectively (Fig. 3d). The SNP and Indel caused the nonsynonymous mutation of genes in HT34, HT36, and HT385, with a number of 10, 13, and 12, respectively (Fig. 3e). Ten mutant genes were both identified in HT34 and HT36, while only one mutant gene (*A005324*) was shared by HT34, HT36, and HT385 (Table 1). Moreover, mutations resulted in the deletion of 2, 3, and 7 genes in HT34, HT36, and HT385, respectively, either by the gain of the stop codon (stopgain) or frameshift. Additionally, abundant SV was found in the chromosome of HT385 compared with that of CA20, especially for the intra-chromosomal translocation (ITX) loci (Fig. 3f).

**Table 1:** Mutated genes of HT34, HT36, and HT385 during thermal adaptive evolution

Gene name	Protein ID	Mutation site	Protein name	Domain^#^	Biological process	Strain
*A000270*	VBB88816	R78C	Hypothetical protein	NA	NA	HT34 and HT36
*A000933*	RDW33678	G231E	Hypothetical protein	Peptidase M28	NA	HT34 and HT36
*A001169*	VBB85575	H230P	Hypothetical protein	Rxt3	Histone deacetylation	HT34 and HT36
*A001179*	RDW29386	K709Q	Nucleoside triphosphate hydrolase	rad25	Nucleotide-excision repair	HT34 and HT36
*A002052*	CAG82270	I273V	YALI0C17655p	NA	NA	HT34 and HT36
*A002823*	VBB88205	M1818I	Regulator of the Gcn2p kinase	Gcn1_N, Adaptin_NM, and HEAT_EZ	Translation regulation	HT34 and HT36
*A002914*	RDW32962	V378A	Hypothetical protein	NA	NA	HT34 and HT36
*A004232*	RDW35484	D229H	DNA-binding factor	GCFC and G-patch	Transcription regulation	HT34 and HT36
*A005324*	RDW50006	A185P	Hypothetical protein	SWIRM	Transcription regulation	HT34, HT36 and HT385
*A005657*	VBB82873	T655S	Hypothetical protein	Peptidase_C19R	Protein deubiquitination	HT34 and HT36
*A000543*	RDW35092	A321T	Hypothetical protein	NA	NA	HT36
*A003076*	VBB88467	Stopgain	Hypothetical protein	NA	Negative regulation of transcription	HT36
*A004804*	CAG83553	Q92K	Phosphatase	MPP_PP2A_PP4_PP6	Mitotic cell cycle	HT36
*A005977*	RDW30514	H121Q	CCAAT-binding transcription factor	CBFB_NFYA	Transcription regulation	HT36
*A001239*	CAG83083	A752V	YALI0B13222p	Pet127	Mitochondrial RNA 5′-end processing	HT385
*A001360*	RDW35242	T128P	Hypothetical protein	NA	NA	HT385
*A001929*	VBB89012	Stopgain	Hypothetical protein	SMC_prok_B	Chromosome segregation	HT385
*A002512*	VBB83323	Q363R	66S pre-ribosomal particles	RRP14	Ribosomal subunit biogenesis	HT385
*A003318*	VBB89475	Q1731L	Hypothetical protein	YCG1, Nipped-B_C, and Amelogenin	Replication-born double-strand break repair via sister chromatid exchange	HT385
*A003822*	RDW29636	Stopgain	Mitochondrial carrier protein	Mito_carr, FRQ1, and EF-hand_7	ATP transport	HT385
*A003939*	RDW35789	D300N	Kinase	Pkc_like	Protein amino acid phosphorylation	HT385
*A003945*	VBB78739	L209Q	Hypothetical protein	HSF_DNA-bind	NA	HT385
*A004345*	VBB77996	F94S	Hypothetical protein	PRK06975, PHA03307, and Smc	Transcription regulation	HT385
*A004506*	RDW30184	Stopgain	Armadillo-type protein	NA	Protein import into nucleus	HT385
*A004754*	RDW34744	V136I	Hypothetical protein	COG5540	Posttranslational modification	HT385
*A004938*	RDW39390	R270H	Mitochondrial carrier protein	PTZ00169	L-glutamate transmembrane transport	HT385
*A005130*	RDW32125	Y254C	IF2B/IF5 protein	W2_eIF5 and eIF-5_eIF-2B	Translational initiation	HT385
*A005261*	RDW30698	S112F	Hypothetical protein	PEX10	Posttranslational modification	HT385
*A001479*	RDW51737	Frameshift deletion	Hypothetical protein	PP2Cc, CYCc, RA_CYR1_like, and PLN00113	Signal transduction	HT385
*A004031*	SEI35917	Frameshift deletion	YALIA101S09e01662g1_1	NA	NA	HT385
*A004520*	RMI98785	Frameshift deletion	Hypothetical protein	F-BAR_PombeCdc15_like, SH3, and PHA03247	Cytoskeleton organization	HT34, HT36 and HT385
*A005494*	SEI32238	Frameshift deletion	YALIA101S02e12772g1_1	PKc_like	Signal transduction	HT34, HT36, and HT385

^#^The domain of each protein was predicted by NCBI Conserved Domain Search (https://www.ncbi.nlm.nih.gov/Structure/cdd/wrpsb.cgi). *NA*, not accessible

Functional annotation of the mutant genes showed that abundant genes in HT34 or HT36 were involved in transcription regulation (e.g., *A003076*, *A004232*, *A001169*, and *A005977*) and translation regulation (*A002823*). The mutant genes in HT385 mainly participated in signal transduction (*A001479*, *A003939*, and *A005494*), translational initiation and posttranslational modification (*A004754*, *A005130*, and *A005261*), and ATP transport (*A003822*). In addition, genes related to DNA repair (*A001179* and *A003318*) and cell cycle (*A001929*) were found to be mutated during adaptive evolution. Collectively, these results suggest that mutation of genes involved in transcription and translation regulation is one important way for cells to adapt to the increased thermal stress.

### Global gene expression of the thermotolerant Y. lipolytica

**Fig. 4:**
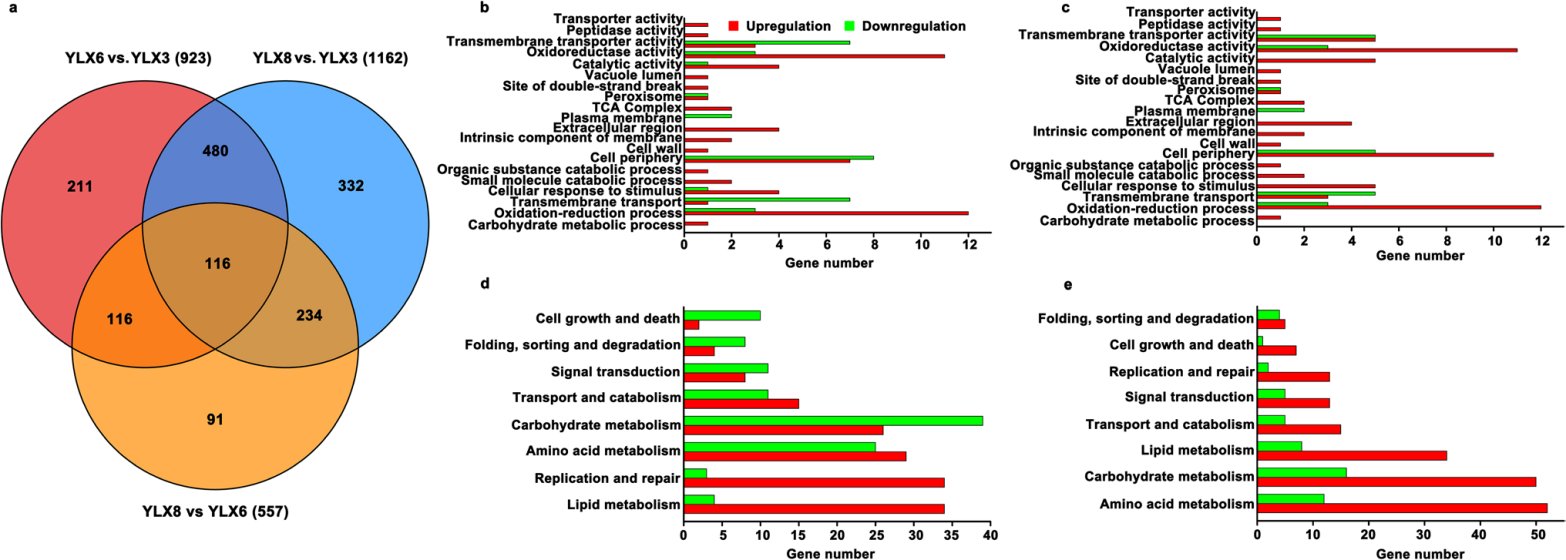
Functional enrichment analysis of the DEGs. **a** Venn diagram among groups YLX6 vs. YLX3, YLX8 vs. YLX3, and YLX8 vs. YLX6. GO enrichment analysis of the DEGs in YLX6 (**b**) and YLX8 (**c**) compared with that in YLX3. KEGG enrichment analysis of the DEGs in YLX6 (**d**) and YLX8 (**e**) compared with that in YLX3

Knowing that genes involved in expression regulation were mutated during thermal adaptive evolution, we sought to deepen our understanding of the thermotolerance mechanism of these strains at a transcriptional level. To achieve this goal, the transcriptomes of *Y. lipolytica* CA20 (named as YLX3), *Y. lipolytica* HT36 (named as YLX6), and *Y. lipolytica* HT385 (named as YLX8) were analyzed and compared. Totally, 923 and 1162 genes were identified to be differentially expressed in YLX6 and YLX8 compared with those in YLX3, of which 653 and 888 genes were found to be upregulated, respectively (Supplemental Fig. S3). Moreover, 557 genes were differentially expressed in YLX8 compared with those in YLX6, wherein 372 genes were upregulated. The number of common DEGs found among groups YLX6 vs. YLX3, YLX8 vs. YLX3, and YLX8 vs. YLX6 was 116 (Fig. 4a and Supplemental Table S3).

The genes encoding hypothetical proteins with unknown functions were not discussed in this study. GO enrichment analysis showed that most of the upregulated genes in YLX6 and YLX8 encode proteins that are located at the cell periphery, extracellular region, intrinsic component membrane, and TCA complex (Fig. 4b, c). These proteins predominantly possess oxidoreductase activity, catalytic activity, and transmembrane transporter activity, functioning in bioprocesses like the oxidation–reduction process, cellular response to stimulus, and small-molecule catabolic process. The downregulated genes in YLX6 and YLX8 mainly code for proteins that are located at the cell periphery and plasma membrane, possessing oxidoreductase activity and transmembrane transporter activity, and functioning in transmembrane transport and the oxidation–reduction process (Fig. 4b, c). KEGG analysis showed that most of the DEGs found in YLX6 and YLX8 were distributed in pathways including carbohydrate metabolism, amino acid metabolism, replication and repair, lipid metabolism, signal transduction, cell growth and death, and transport and catabolism (Fig. 4d, e). We next conducted a detailed study of the transcriptional changes of these important pathways and within the functional groups revealed by the enrichment analysis.

### Cell envelope structures

**Fig. 5:**
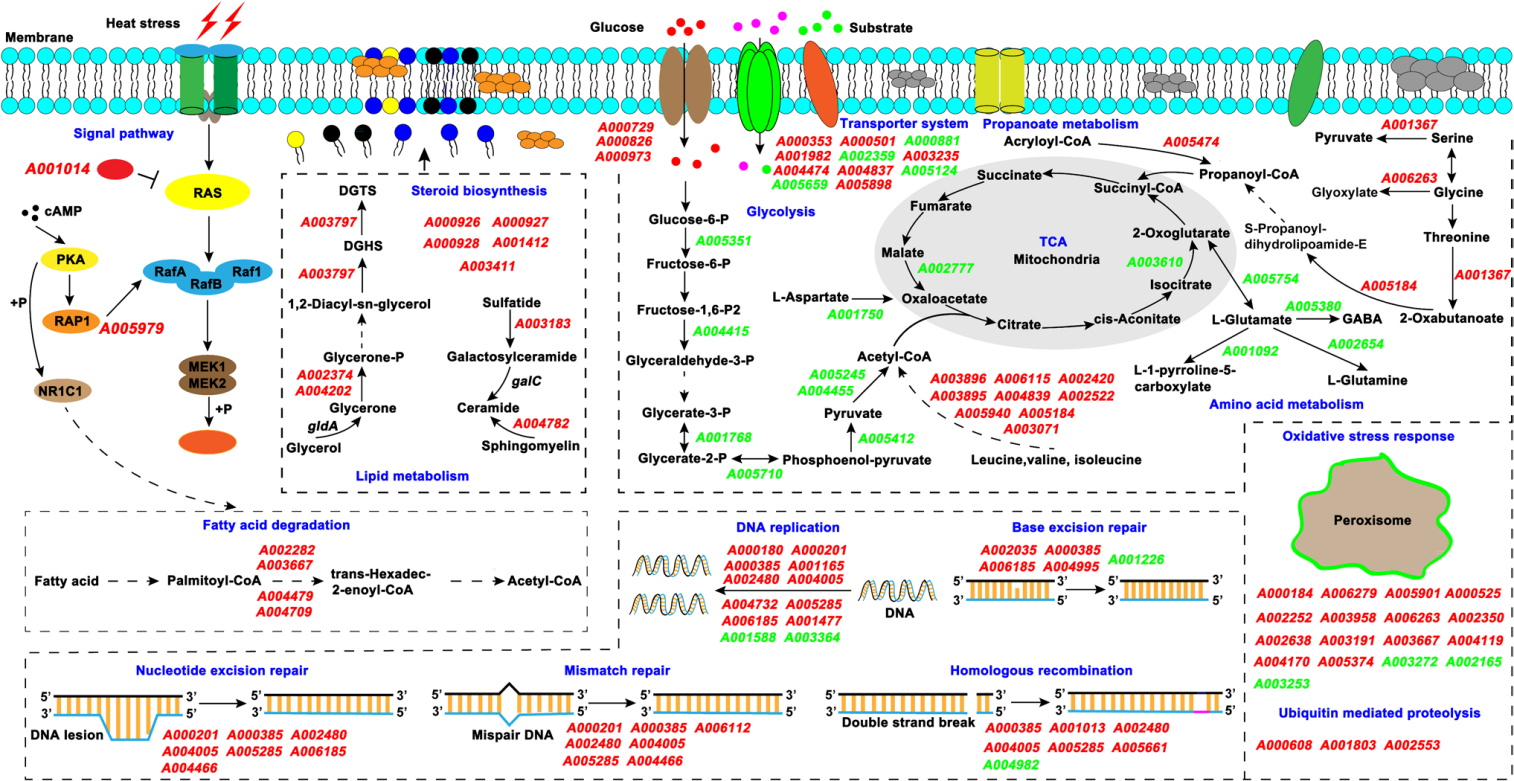
Outline of the DEGs in YLX6 and YLX8 compared with those in YLX3. DEGs were shown to be involved in biological processes including signal pathway, lipid metabolism, fatty acid degradation, DNA replication and repair, transporter system, glycolysis, tricarboxylic acid cycle (TCA), propanoate metabolism, amino acid metabolism, oxidative stress response, and ubiquitin-mediated proteolysis. PKA, cAMP-protein kinase A; RAP1, RAS-related protein Rap-1A; RafA, A-Raf proto-oncogene serine/threonine-protein kinase; RafB, B-Raf proto-oncogene serine/threonine-protein kinase; Raf1, RAF proto-oncogene serine/threonine-protein kinase; MEK1/MEK2, mitogen-activated protein kinase 1/2; NR1C1, peroxisome proliferator-activated receptor alpha; *gldA* encodes glycerol dehydrogenase; DGHS, diacylglycerylhomoserine; DGTS, diacylglyceryl-N,N,N-trimethylhomoserine; *galC* encodes galactosylceramidase; GABA, γ-aminobutyric acid; P, phosphate. An arrow with a dashed line indicates that the corresponding process includes more than one step. The gene names with red and green text indicate upregulation and downregulation, respectively. Detailed Log_2_FC (fold change) values, together with the gene annotations, were listed in the Supplemental Table S4

Membrane channels like the major facilitator superfamily (MFS) transporter and porin are critical for nutrient uptake. We found that 14 DEGs were related to the transporters (Fig. 5 and Supplemental Table S4), of which 10 genes coding for MFS-type transporter (*A000353*, *A003235*, *A004474*, *A004837*, and *A005898*), general substrate transporter (*A000729*), plasma membrane low glucose sensor (*A000826*), amino acid permease (*A000501*), monocarboxylate/proton symporter (*A001982*), and YALIA101S01e17150g1_1 (*A000973*) were upregulated in YLX8 or YLX6 compared with that in YLX3. These changes suggested that cells increased nutrient uptake to defend against thermal stress. The downregulated transporters were correlated with the transport of irons (*A000881*, *A005124*, and *A005659*) and water (*A002359*).

In addition to the transporters, thermal stress also caused the differential expression of genes involved in lipid metabolism (Fig. 5). Notably, the DEGs involved in steroid biosynthesis (*A000926*, *A000927*, *A000928*, *A001412*, and *A003411*), glycerolipid and glycerophospholipid metabolism (*A002374*, *A003797*, and *A004202*), and ceramide synthesis (*A003183* and *A004782*) were upregulated in YLX6 and YLX8 compared with that in YLX3, suggesting that alteration of the membrane lipid component was one important way for cells to grow under thermal stress.

### Glycolysis and tricarboxylic acid cycle

Ten DEGs were identified to be related to glycolysis and the TCA cycle (Fig. 5). Interestingly, the expression of genes coding for phosphoglucose isomerase (*A005351*), fructose-1, 6-bisphosphate (FBP) aldolases (*A004415*), tetrameric phosphoglycerate mutase (*A001768*), enolase (*A005710*), pyruvate kinase (*A005412*), pyruvate dehydrogenase beta subunit (*A004455*), and TPP-dependent 2-oxoacid decarboxylase (*A005245*) decreased in YLX6 compared with that in YLX3 (Supplemental Table S4), suggesting that the glycolysis was inhibited upon thermal stress. Moreover, the expression of genes related to the TCA cycle, including *A002777* and *A003610* coding for malate dehydrogenase and isocitrate dehydrogenase, respectively, was also downregulated in YLX6 compared with that in YLX3, indicating a suppressed TCA cycle of cells upon thermal stress.

### Amino acid metabolism, fatty acid degradation, and propanoate metabolism

The inhibited glycolysis might result in the reduced synthesis of acetyl-CoA, thereby influencing the TCA cycle. Cells might metabolize other molecules to supply acetyl-CoA and maintain the running of the TCA cycle. We found that 16 DEGs were involved in the amino acid metabolism and propanoate metabolism, of which transcriptional upregulation mainly occurred in the genes related to the metabolism of leucine, valine, and isoleucine (*A002420*, *A002522*, *A003071*, *A003895*, *A003896*, *A004839*, *A005184*, *A005940*, and *A006115*), the metabolism of glycine, threonine, and serine (*A001367* and *A006263*), and the metabolism of propanoate (*A005184* and *A005474*) in YLX6 and YLX8 compared with that in YLX3 (Fig. 5 and Supplemental Table S4). Besides, four genes involved in fatty acid degradation (*A002282*, *A003667*, *A004479*, and *A004709*) were shown to be upregulated upon thermal stress. In addition, the expression of genes involved in glutamate metabolism was downregulated in YLX6 and YLX8 compared with that in YLX3, including *A001092*, *A002654*, *A005380*, and *A005754*, suggesting an accumulation of glutamate in the cells. These results suggest that cells metabolize leucine, valine, isoleucine, fatty acid, and propanoate to supply acetyl-CoA and propanoyl for the normal running of the TCA cycle while accumulating glutamate to defend against thermal stress.

### DNA replication, repair, and recombination

Maintenance of genome stability is important for cells to survive in stressful environments. Notably, we found that among the 12 DEGs related to DNA replication, 10 genes (*A000180*, *A000201*, *A000385*, *A001165*, *A001477*, *A002480*, *A004005*, *A004732*, *A005285*, and *A006185*) mainly coding for nucleoside triphosphate hydrolase, DNA polymerase, replication factor, and DNA primase were upregulated in YLX6 and YLX8 compared with that in YLX3 (Fig. 5 and Supplemental Table S4). Some of these genes were also involved in DNA repair. Additionally, transcriptional upregulation was observed for the genes included in nucleotide excision repair (*A004466*), mismatch repair (*A004466* and *A006112*), base excision repair (*A002035* and *A004995*), and homologous recombination (*A001013* and *A005661*). These results suggest that cells increase genome stability to survive under thermal stress.

### Oxidative stress response and signaling pathway

To withstand the oxidative stress caused by high temperature, cells increased the expression of genes involved in peroxisome. Among the 17 DEGs, the expression of 14 genes coding for hydrolase protein (*A000184*, *A004170*, and *A005374*), peroxisome biogenesis factor (*A000525* and *A002350*), catalase (*A006279*), choline/carnitine o-acyltransferase (*A005901*), deoxycytidine monophosphate (dCMP) deaminase (*A002252*), peroxisomal 2, 4-dienoyl-CoA reductase (*A003958*), FAD/FMN-dependent oxidoreductase (*A002638* and *A006263*), peroxisome assembly protein 12 (*A003191*), acyl-CoA oxidase (*A003667*), and peroxisomal 3-oxoacyl-CoA thiolase (*A004119*) was upregulated, while 3 genes coding for catalase (*A003253* and *A003272*) and redoxin (*A002165*) were downregulated (Fig. 5 and Supplemental Table S4). Moreover, the expression of genes coding for WD40-repeat-containing protein (*A000608*) and ubiquitin-conjugating enzyme (*A001803* and *A002553*) was upregulated, indicating that oxidative stress induced protein aggregation and misfolding. The RAS-controlled signal pathway (cAMP-protein kinase A (PKA) pathway) plays a positive role in the control of dimorphic transition in yeast (Liang et al. 2017). We found that the expression of two genes coding for RAS-related proteins (*A001014* and *A005979*) was increased in YLX6 and YLX8 compared with that in YLX3, suggesting an involvement of the RAS-cAMP-PKA signal pathway in the thermal stress response.

### Erythritol metabolism

**Fig. 6:**
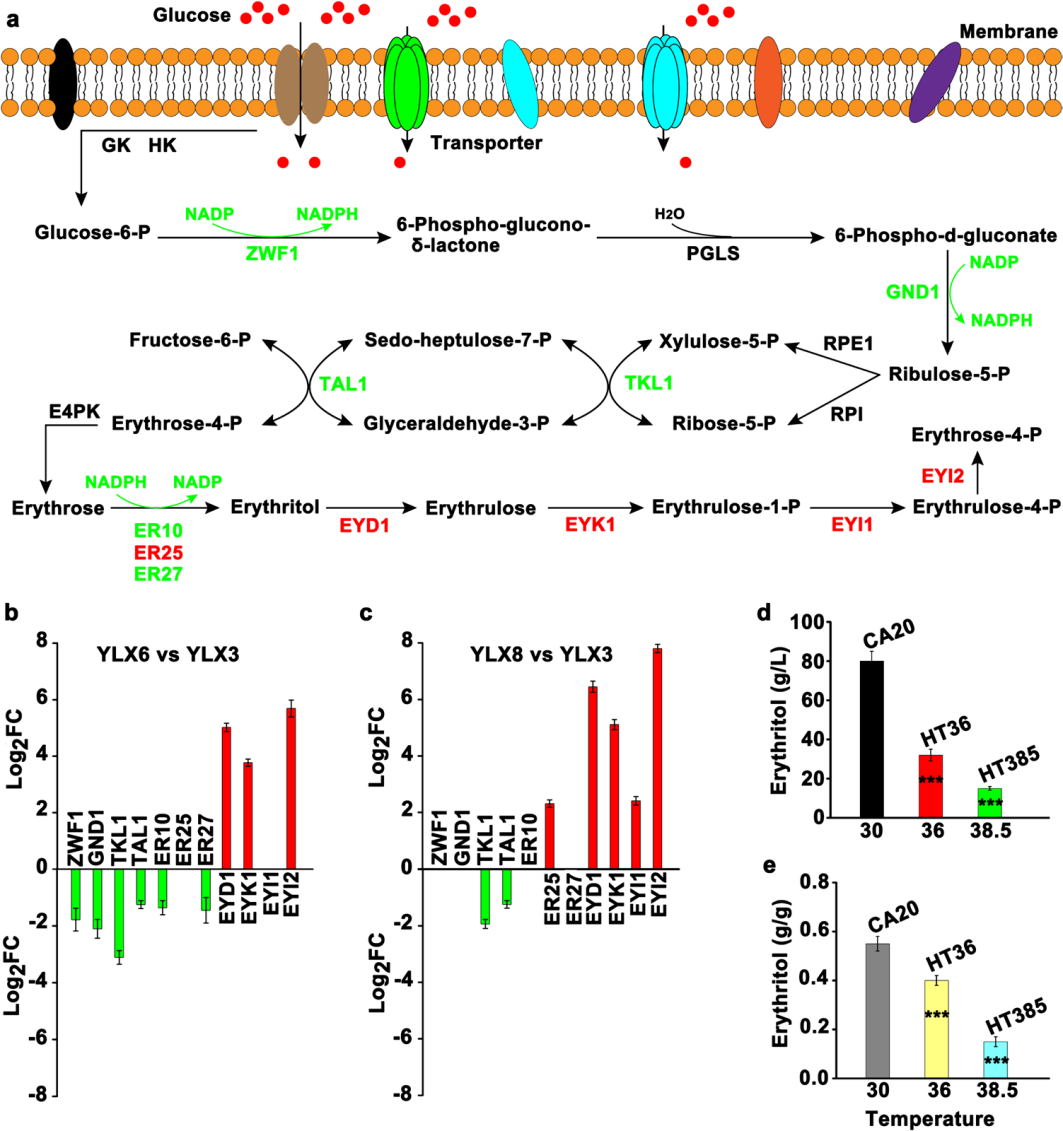
DEGs involved in erythritol metabolism. **a** Erythritol metabolism pathway. **b** Changes in the expression level of genes in YLX6 compared with that of YLX3. **c** Changes in the expression level of genes in YLX8 compared with that of YLX3. **d** The production of erythritol by *Y. lipolytica* CA20, *Y. lipolytica* HT36, and *Y. lipolytica* HT385 at different temperatures. **e** The erythritol yield. GK, glucokinase; HK, hexokinase; ZWF1, glucose-6-phosphate dehydrogenase; PGLS, 6-phosphogluconolactonase; GND1, phosphogluconate dehydrogenase; RPE1, ribulose phosphate 3-epimerase; RPI, ribulose-5-phosphate isomerase; TKL1, transketolase; TAL1, transaldolase; E4PK, erythrose-4-phosphate kinase; ER, erythrose reductase; EYD1, erythritol dehydrogenase; EYK1, erythrulose kinase; EYI1, erythrulose-1-phosphate isomerase; EYI2, erythrulose-4-phosphate isomerase; P, phosphate. The green and red text represent downregulation and upregulation, respectively. Data obtained from three biological replicates were shown as the mean ± standard deviation. “*” indicates that there is a significant difference between the two studied groups (***p < 0.001), in which CA20 is set as the control group

Erythritol could be synthesized from glucose through the pentose phosphate pathway (Fig. 6a). Here, we found that 11 DEGs were involved in erythritol metabolism, among which *A004280* coding for glucose-6-phosphate dehydrogenase (ZWF1), *A001342* coding for phosphogluconate dehydrogenase (GND1), *A003663* coding for transketolase (TKL1), *A005663* coding for transaldolase (TAL1), *A005140*, *A001913*, and *A005791* coding for erythrose reductase (ER10, ER25, and ER27) were responsible for erythritol synthesis, while *A002376* coding for erythritol dehydrogenase (EYD1), *A002374* coding for erythrulose kinase (EYK1), *A002373* coding for erythrulose-1-phosphate isomerase (EYI1), and *A002375* coding for erythrulose-4-phosphate isomerase (EYI2) were involved in erythritol degradation (Fig. 6a and Supplemental Table S4). Notably, almost all of the genes involved in erythritol synthesis were downregulated in YLX6 and YLX8 compared with those in YLX3, while the genes related to erythritol degradation were upregulated, suggesting a decreased accumulation of erythritol in HT36 and HT385 (Fig. 6b, c). Expectedly, the titer and yield of erythritol were significantly decreased in HT36 and HT385 compared with those of CA20 (Fig. 6d, e).

### Alteration of SOD and CAT activity, the content of intracellular FFA, AA, and ATP in Y. lipolytica HT385

**Fig. 7:**
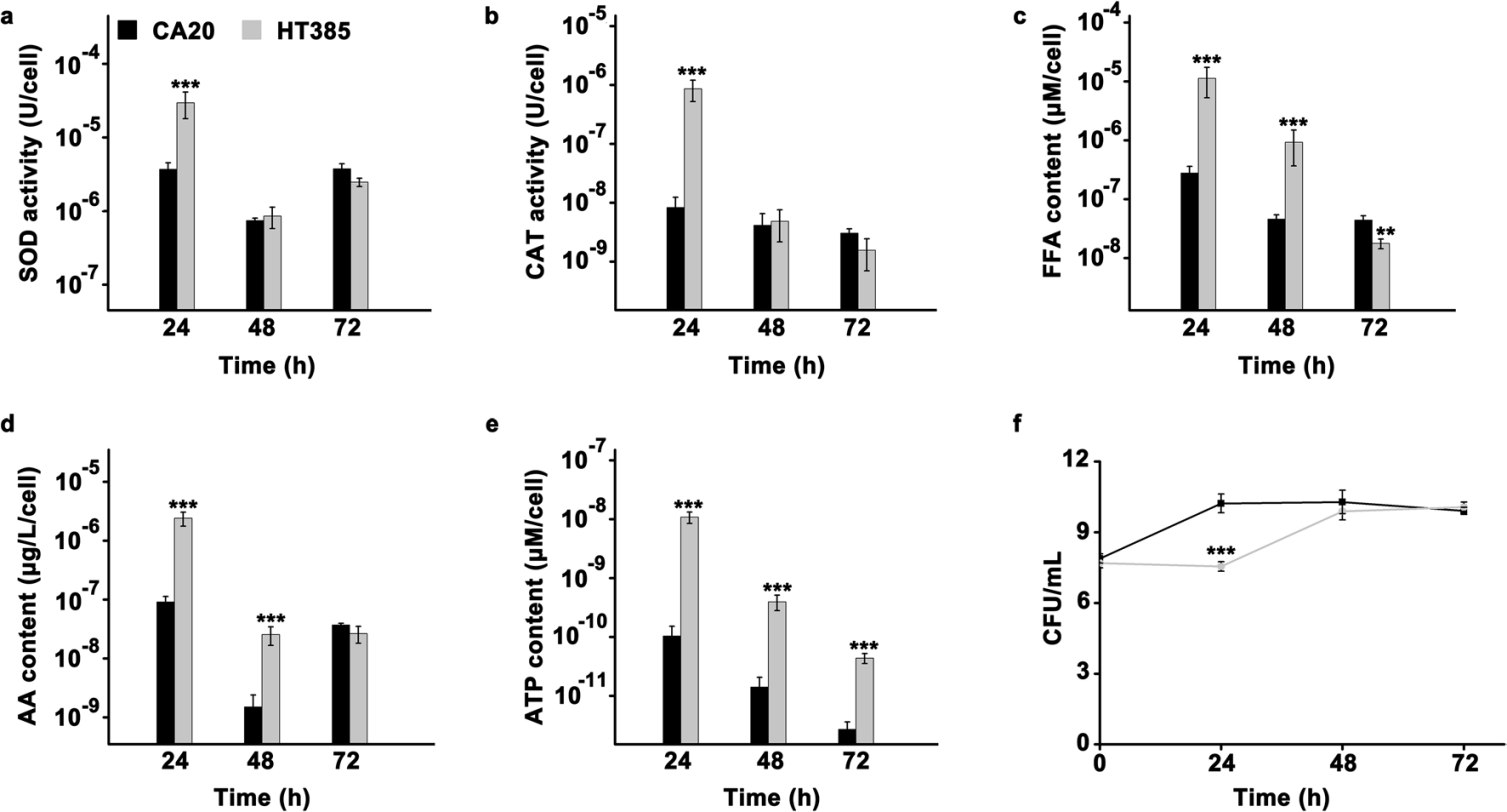
Alteration of the SOD activity (**a**), CAT activity (**b**), FFA content (**c**), AA content (**d**), ATP content (**e**), and CFU (**f**) of *Y. lipolytica* HT385 and *Y. lipolytica* CA20 throughout the whole growth phase. Data obtained from three biological replicates were shown as the mean ± standard deviation. “*” indicates that there is a significant difference between the two studied groups (**p < 0.01; ***p < 0.001). SOD, superoxide dismutase; CAT, catalase; FFA, free fatty acid; AA, amino acid; CFU, colony-forming units

SOD and CAT are essential for cells to defend against oxidative stress. Interestingly, at a transcriptional level, the genes coding for SOD were not found to be differentially expressed in YLX8 compared with that in YLX3, while two of the three DEGs coding for catalase were downregulated (Supplemental Table S4). We next detected the SOD and CAT activity of *Y. lipolytica* HT385 and *Y. lipolytica* CA20 throughout the growth phase. The results showed that the SOD activity of HT385 was significantly higher than that of CA20 when grown for 24 h, after which the SOD activity of HT385 and CA20 decreased as cells grew, and there was no significant difference observed between the two groups at 48 and 72 h (Fig. 7a). A similar phenomenon was observed for the changes of CAT activity in HT385 and CA20 (Fig. 7b). These findings overall supported the results obtained from the transcriptome analysis.

For the changes of FFA content, we found that the FFA content of HT385 and CA20 decreased gradually as cells grew, with the FFA content of HT385 being significantly higher than that of CA20 within 48 h while lower than that of CA20 at 72 h (Fig. 7c). Similarly, the AA content of HT385 decreased as cells grew and was significantly higher than that in CA20 within 48 h (Fig. 7d). These results appeared to support the conclusion deduced from the transcriptome analysis that cells metabolized FFA and AA to increase the synthesis of acetyl-CoA to defend against thermal stress. Additionally, the ATP content of HT385 was significantly higher than that in CA20 throughout the growth phase (Fig. 7e), emphasizing the importance of ATP in the thermal stress response. CFU detection showed that the cell number of HT385 was lower than that of CA20 at 24 h (Fig. 7f), which was consistent with the growth curve of HT385 at 38.5 °C, wherein a lag phase was observed within 24 h (Fig. 2d).

### Influence of glycerol, soybean oil, trehalose, AA, thiamine, and ATP on the growth of Y. lipolytica HT385

**Fig. 8:**
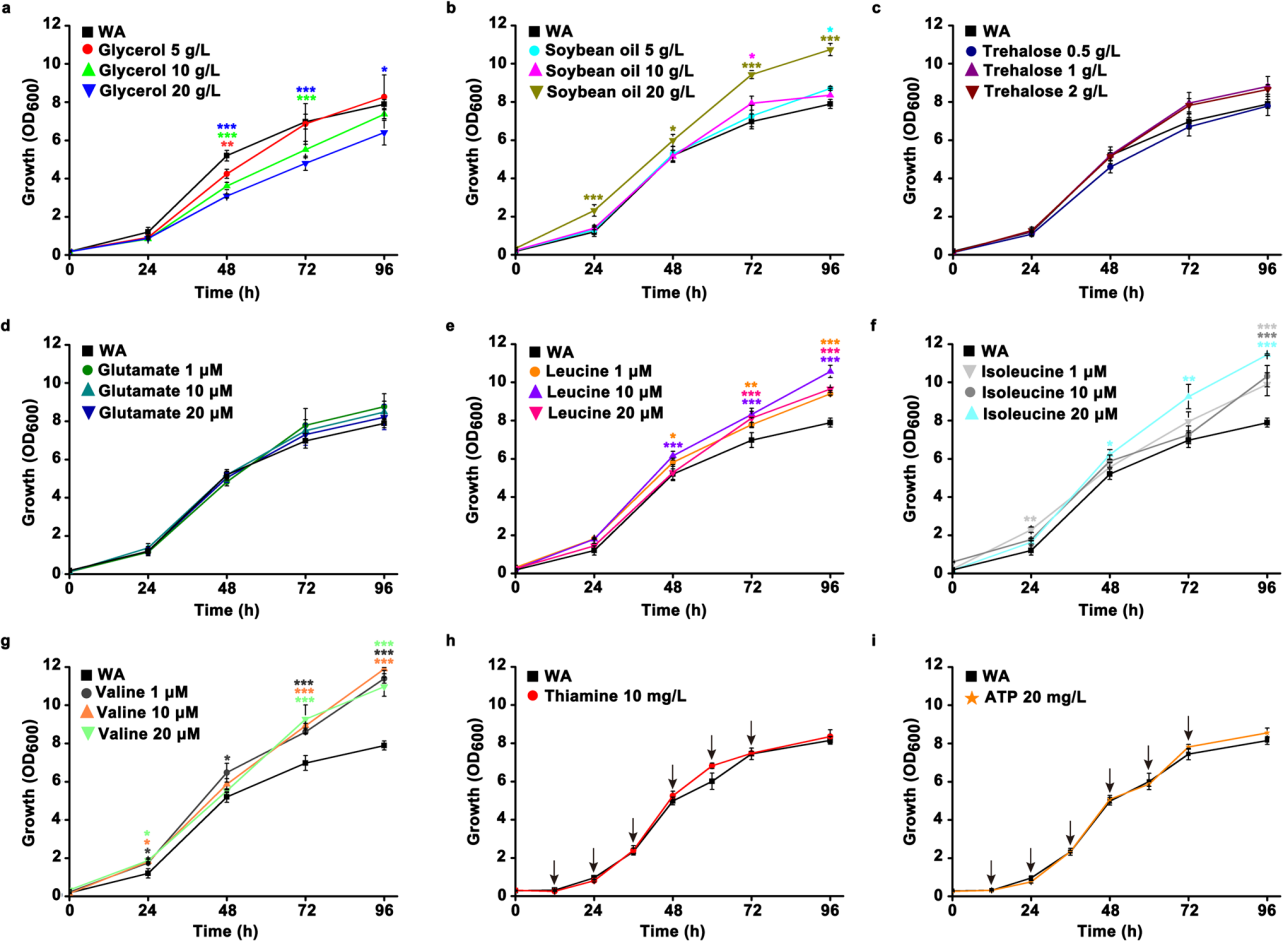
The influence of glycerol (**a**), soybean oil (**b**), trehalose (**c**), glutamate (**d**), leucine (**e**), isoleucine (**f**), valine (**g**), thiamine (**h**), and ATP (**i**) on the growth of *Y. lipolytica* HT385. Data obtained from three biological replicates were shown as the mean ± standard deviation. Different colors of “*” indicating significant differences are in accordance with the respective strain to the control group (*p < 0.05; **p < 0.01; ***p < 0.001). WA, without addition; arrows indicate that the thiamine or ATP is added at the corresponding time points

After knowing that FFA and AA metabolism contributed to the thermotolerance of *Y. lipolytica* HT385, we sought to figure out whether the exogenous addition of these substances could enhance the growth of cells. Meanwhile, the effect of glycerol, thiamine, trehalose, and ATP on the growth of HT385 was determined as well. The results showed that the addition of glycerol significantly suppressed the growth of HT385 compared with the control group (WA), and the inhibitory effect was enhanced as the glycerol concentration increased (Fig. 8a). Soybean oil contains abundant fatty acids. It was found that addition of soybean oil with a final concentration of 5 or 10 g/L produced little influence on the cell growth within 48 h, whereas soybean oil of 20 g/L significantly promoted the cell growth compared with that of WA and also shortened the lag phase within 24 h (Fig. 8b). The addition of trehalose with a final concentration of either 0.5, 1, or 2 g/L had little effect on the growth of cells (Fig. 8c). Similar results were observed for the addition of glutamate, wherein 1, 10, and 20 μM glutamate were not able to promote cell growth (Fig. 8d). In addition, it was worth noting that the addition of either leucine, isoleucine, or valine could significantly promote cell growth compared with that of WA in most cases (Fig. 8e–g). Moreover, the addition of leucine, isoleucine, and valine could increase the maximum cell density. Additionally, we found that the addition of thiamine or ATP with an interval of 12 h was not able to facilitate cell growth (Fig. 8h, i). Collectively, these results further support that FFA, leucine, isoleucine, and valine are critical for cells to grow under thermal stress.

### Effect of the overexpression of upregulated genes on the growth and erythritol production of Y. lipolytica CA20

**Fig. 9:**
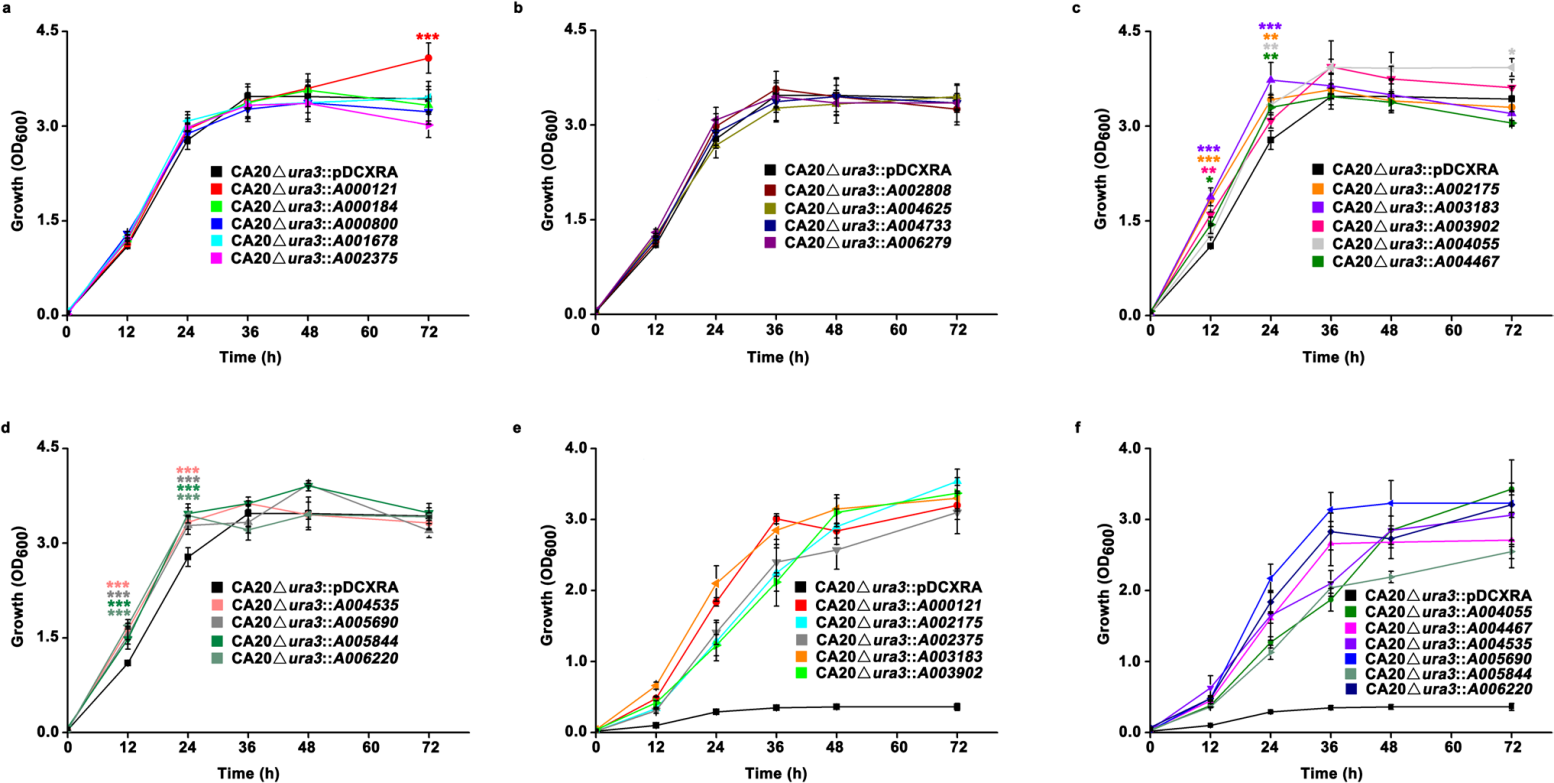
Growth curves of CA20∆*ura3* with an overexpression of the upregulated genes at 30 °C (**a**–**d**) and 34 °C (**e**, **f**). The YNBD medium was used in the growth assay. Data obtained from three biological replicates were shown as the mean ± standard deviation. Different colors of “*” indicating significant differences are in accordance with the respective strain to the control group (*p < 0.05; **p < 0.01; ***p < 0.001). The comparison was conducted between the CA20∆*ura3* harboring a target gene and the vector pDCXRA

Transcriptome analysis showed that abundant genes coding for hypothetical proteins with unknown functions were upregulated in thermotolerant strains, indicating the important roles of these genes in the thermal stress response. To test this hypothesis, eighteen representative genes with a relatively higher expression level were selected and overexpressed in *Y. lipolytica* CA20 (Supplemental Table S5). Among the eighteen genes, three were related to ceramide synthesis (*A003183*) and the peroxisome (*A000121* and *A006279*), while other genes mainly encode hypothetical proteins. We found that overexpression of either *A000184*, *A000800*, *A001678*, *A002375*, *A002808*, *A004625*, *A004733*, or *A006279* had little effect on the growth of CA20 at 30 °C, as no significant difference in cell growth was observed between the CA20∆*ura3* harboring a target gene and the vector pDCXRA throughout the growth phase (Fig. 9a, b). On the contrary, individual overexpression of the genes *A002175*, *A003183*, *A003902*, *A004055*, *A004467*, *A004535*, *A005690*, *A005844*, and *A006220* significantly promoted cell growth within 24 h compared with that observed in CA20∆*ura3*::pDCXRA (Fig. 9c, d). In addition, overexpression of *A000121* improved cell growth after 48 h.

Further, we investigated whether overexpression of the selected genes could enable cells to grow at a temperature higher than 30 °C. It was found that overexpression of *A000121*, *A002175*, *A002375*, *A003183*, *A003902*, *A004055*, *A004467*, *A004535*, *A005690*, *A005844*, and *A006220* individually enabled the cells to grow at 34 °C, while CA20∆*ura3* harboring the vector pDCXRA was not able to grow (Fig. 9e, f). Notably, CA20∆*ura3* with an overexpression of either *A000121*, *A003183*, or *A005690* grew better than other strains at 34 °C, suggesting that these genes would be good candidates for the construction of thermotolerant strains by genetic engineering. Further, the growth of these strains at 35, 36, and 38.5 °C was tested. It was found that cells with an overexpression of these genes were not able to grow at 35 °C (Supplemental Fig. S4) or higher temperatures (data not shown).

**Fig. 10:**
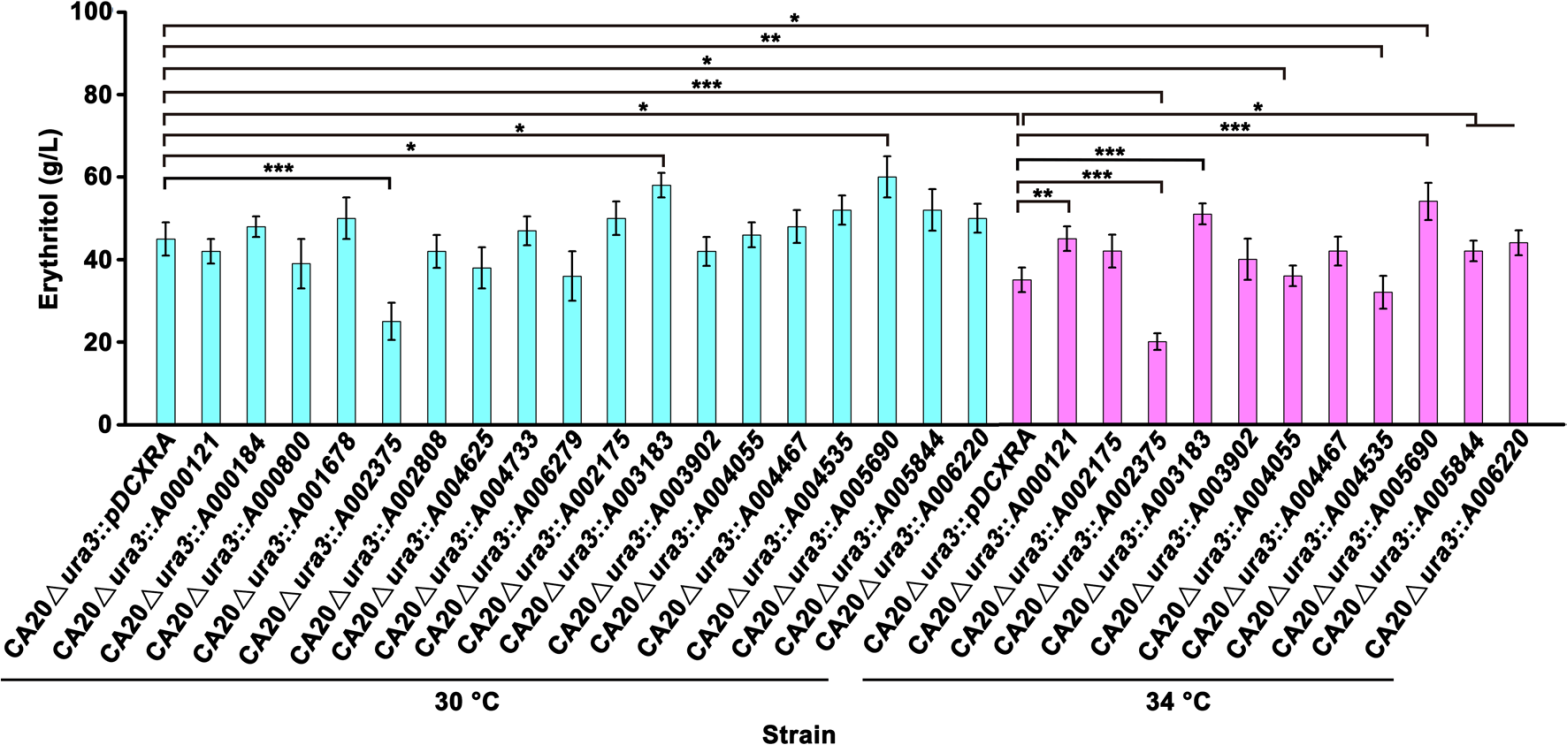
Effect of overexpression of the upregulated genes on erythritol production by CA20∆*ura3* at 30 °C and 34 °C. The erythritol production was carried out using YNBD medium containing 200 g/L glucose. Data obtained from three biological replicates were shown as the mean ± standard deviation. “*” indicates a significant difference (*p < 0.05; **p < 0.01; ***p < 0.001)

Effect of overexpression of the upregulated genes on erythritol production at 30 and 34 °C was further investigated. The results showed that individual overexpression of the most genes had little effect on the erythritol production by CA20∆*ura3* at 30 °C, as no significant difference in the erythritol titer was observed between the CA20∆*ura3* harboring a target gene and the vector pDCXRA (Fig. 10). Overexpression of *A002375* significantly decreased the erythritol titer, while individual overexpression of *A003183* and *A005690* significantly promoted the synthesis of erythritol by CA20∆*ura3*. At 34 °C, the production of erythritol was inhibited in CA20∆*ura3*::pDCXRA compared with that obtained at 30 °C. Similarly, overexpression of *A002375* also significantly decreased the erythritol titer. Nevertheless, the erythritol production of CA20∆*ura3* with an individual overexpression of *A000121*, *A003183*, *A005844*, and *A006220* was significantly increased compared with that of CA20∆*ura3*::pDCXRA. The erythritol titers of these strains obtained at 30 °C and 34 °C were similar, as no significant difference was observed. Moreover, the erythritol titer of CA20∆*ura3*::*A005690* was increased and even significantly higher than that obtained at 30 °C (Fig. 10). Collectively, these results suggest that overexpression of some upregulated genes could not only endow cells with thermotolerance but also preserve erythritol production.

## Discussion

### ALE combined with mutagenesis is an efficient way to breed thermotolerant Y. lipolytica

In the past years, ALE has been a valuable tool for improving the microbial phenotypes to meet production requirements, through which strains with improved tolerance to thermal stress, acid stress, and inhibitors from lignocellulosic biomass have been obtained in *S. cerevisiae* (Sandberg et al. 2019; Mavrommati et al. 2022; Salas-Navarrete et al. 2023). For *Y. lipolytica*, Wang and co-authors used ALE to enhance the ferulic acid tolerance of *Yarrowia lipolytica* XYL + , a strain that was sensitive to 0.5 g/L ferulic acid (Wang et al. 2021). Finally, it took 84 days for the whole ALE, and the obtained cells could survive well in medium with 1.5 g/L ferulic acid. In another study, Zhou and co-authors adopted ALE to improve the aromatic aldehydes tolerance of *Y. lipolytica* XYL + (Zhou et al. 2021). They showed that it took about 20–30 days for XYL + to obtain tolerance to 0.50 g/L vanillin, syringaldehyde, or 4-hydroxybenzaldehyde and 70 days to obtain tolerance to 0.75 g/L 4-hydroxybenzaldehyde. Daskalaki and co-authors used ALE to increase the lipid accumulation of* Yarrowia lipolytica* ACA DC 50109, in which a population obtained after 77 generations was able to accumulate 44% (w/w) of lipid, with a 30% higher than that of the starting strain (Daskalaki et al. 2019). Nevertheless, to the best of our knowledge, the breeding of erythritol-producing *Y. lipolytica* capable of growing at a temperature higher than 37 °C has rarely been reported until now. Qiu and co-authors obtained a thermotolerant *Yarrowia lipolytica* BBE-18 by ALE from a start strain BBE-17 grown at 30 °C (Qiu et al. 2021). They showed that the OD and specific growth rate of BBE-18 at 35 °C were similar to those of BBE-17 at 30 °C. Nonetheless, the strain BEE-18 was obtained after 11 months of continuous cultivation and selection (Qiu et al. 2021), obviously longer than the time needed for the obtainment of tolerant strains to other stresses as mentioned above, suggesting that the breeding of thermotolerant strains capable of synthesizing erythritol is a time-consuming work.

The long time needed for the breeding of thermotolerant strains might result from two aspects. First, unlike directed evolution, ALE gives new phenotypes via random mutation, which requires extended time for selection (Sandberg et al. 2019). Second, high osmotic stress maintained by the addition of glucose with a high concentration is necessary for the synthesis of erythritol by *Y. lipolytica*, which will inevitably influence the adaptation of cells to thermal stress. To overcome this deficiency, we attempted to use mutagenesis integrated with ALE to increase the mutation rate, thereby accelerating the execution of ALE. Referring to previous studies, atmospheric and room temperature plasma (ARTP) mutagenesis combined with ALE was shown to be a promising way for improving microbial performance, of which ARTP could induce DNA leakage and recombination (Cui et al. 2018; Liu et al. 2020). However, whether ARTP combined with ALE is an efficient way for breeding thermotolerant strains remains elusive. In the preliminary experiments, we found that the effect of the use of ARTP integrated with ALE was not better than the independent use of ALE on the selection of thermotolerant strains (data not shown). In our previous study, we revealed that ^60^Co-γ radiation was efficient for the breeding of erythritol-high-producing strains (Liu et al. 2023). Hence, we tried ^60^Co-γ radiation and UV radiation in this study. Additionally, the protectant trehalose was added during the breeding process to help cells adapt to thermal stress. As expected, we obtained thermotolerant strains within 150 days (corresponding to 352 generations), shorter than that recorded in the previous study (Qiu et al. 2021). Furthermore, we determined that the thermotolerant phenotype was stable rather than tentative. Collectively, ALE combined with mutagenesis is suggested to be an efficient way for breeding thermotolerant *Y. lipolytica* used in erythritol production.

### Cells tune gene expression by mutating global regulators to adapt to thermal stress

Unraveling the genome changes during adaptive evolution is helpful for clarifying the genetic basis of the target phenotype. Currently, the gene mutations of *Y. lipolytica* during thermal adaptive evolution have not been fully investigated. The knowledge of this aspect predominantly comes from the studies of *S. cerevisiae*. By comparative genomics analysis, previous works showed that genes coding for the elements of RAS-cAMP-PKA and SNF1-GAL83 signaling pathways (*RAS2*, *GPA2*, *ASC1*, *IRA2*, *REG1*, *CAT8*, *HSF1*, and *MIG2*) were mutated in thermotolerant strains (Salas-Navarrete et al. 2021, 2023). Another study presented that the evolved thermotolerant strain capable of growing at 42 °C contained mutations in genes *STE11*, *IRA2*, *CDC25*, *CYR1*, *PKH1*, *STT4*, *EFR3*, *LRG1*, and *CRZ1* that are involved in stress response networks, including the Hog1-mediated pathway, the RAS-cAMP pathway, and the Rho1-Pkc1-mediated cell wall integrity pathway (Huang et al. 2018; Mavrommati et al. 2022). Furthermore, *S. cerevisiae* grown at 40 °C mutated genes mainly related to membrane composition, respiration, DNA repair and replication, ATP synthesis, and 60S ribosome subunit synthesis (Caspeta et al. 2014). In line with their results, we showed that genes related to DNA repair (*A001179* and *A003318*) and ATP transport (*A003822*) were mutated in *Y. lipolytica* during adaptive evolution (Table 1). Otherwise, abundant genes with nonsynonymous mutation sites coding for transcription and translation regulators were newly found in HT34, HT36, and HT385.

Among the mutated genes related to transcription, the genes *A001169*, *A002823*, *A00597*, *A003939*, and *A005494*, coding for an Rxt3-domain-containing protein, a regulator of the Gcn2p kinase, a CCAAT-binding transcription factor, and Pkc domain-containing proteins, are noteworthy. Rxt3 is one component of the large Rpd3 histone deacetylation complex, which has been proven to participate in modulating the expression of SNF1/AMPK-dependent respiratory genes (Tamari et al. 2016). The Gcn2p kinase is involved in the nutritional stress response through the phosphorylation of eukaryotic initiation factor-2 (eIF2) and the general amino acid control pathway (Zaborske et al. 2009). The CCAAT-binding transcription factor has been previously revealed to have roles in regulating sterol metabolism and the transition between fungal hyphal growth and asexual reproduction in *Aspergillus fumigatus* (Ren et al. 2021). Moreover, it is clear that Pkc1 plays roles in many cellular processes, including the maintenance of cell wall integrity by regulating a mitogen-activated protein (MAP) kinase cascade (Dickson et al. 2006; Canonero et al. 2022). Thus, the mutation of these genes may change their activity, thereby affecting the expression of downstream genes.

Finally, we found that nearly all of the identified mutated genes were not differentially expressed upon thermal stress, indicating the different thermotolerance mechanisms of *Y. lipolytica* at genomic and transcriptional levels. Collectively, these findings suggest that cells tune gene expression by changing the activity of global regulators to adapt to thermal stress rather than mutation of the genes involved in specific metabolic processes.

### Cells strengthen genome stability, the degradation of BCAA and erythritol, and ceramide synthesis to defend against thermal stress

Current knowledge of the thermotolerance mechanism of *Y. lipolytica* dependent on transcriptome analysis could be mainly divided into aspects including that cells upregulate the expression of genes responsible for trehalose and alpha ketoglutaric acid accumulation, ATP synthesis, oxidative stress response, heat shock response, central carbon metabolism, amino acid metabolism (alanine, arginine, asparagine, glutamine, methionine, proline, and histidine), and thiamine metabolism (Qiu et al. 2021; Celinska 2022). Thus, it is expected that the differential expression of genes related to these processes will be identified in this study. However, except for the genes involved in oxidative stress response, the genes related to trehalose, ATP, and thiamine synthesis were not found to be differentially expressed in HT36 and HT385 compared with those in CA20. This contradiction may result from that these genes may undergo posttranscriptional modification, as the ATP content of HT385 is observed to be significantly higher than that of CA20. Further, we investigated the influence of trehalose, ATP, and thiamine on the growth of HT385. Unexpectedly, the exogenous addition of these substances had little effect on the growth of HT385. Especially, the addition of ATP was not able to promote cell growth throughout the growth phase, which contradicted the result displayed in the previous study, wherein the addition of exogenous ATP at a dose of 30 nM could promote the rapid growth of cells in the later stage (Qiu et al. 2021). This might be explained by the different conditions and strains used in the two studies. Alternatively, it may result from the complicated thermotolerance mechanisms of *Y. lipolytica*. Further studies are needed to clarify this.

Regardless of the contradiction with the previous study, we found that cells increased genome stability to survive thermal stress as abundant genes related to DNA repair were upregulated. Moreover, genes involved in DNA repair were mutated during adaptive evolution. This was also supported by the genomic analysis, wherein the number of SNP decreased in HT385 compared with that of HT34 and HT36. Maintenance of genome stability is thus suggested to be necessary for the protein synthesis and cell proliferation of HT385. Moreover, we found that thermal stress inhibited glycolysis, probably leading to the impaired synthesis of acetyl-CoA upon thermal stress. Meanwhile, the upregulation of genes related to BCAA (leucine, isoleucine, and valine) and fatty acid degradation suggests that cells metabolize these substances to supply acetyl-CoA, thereby maintaining the normal running of cell proliferation. A promoted growth of HT385 upon exogenous addition of leucine, isoleucine, valine, and soybean oil supports this hypothesis, highlighting the importance of BCAA and FFA on cell fitness under thermal stress.

The inhibited synthesis of erythritol under thermal stress is a common phenomenon found in previous studies, which is ascribed to the decreased expression of GND1, ER25, ER27, and TKL1 at a transcriptional level (Qiu et al. 2021; Zhang et al. 2022). In accordance with their findings, the expression of GND1, ER27, and TKL1 was observed to be downregulated in HT36 and HT385 compared with that in CA20. Moreover, the downregulated expression of genes coding for ZWF1, TAL1, and ER10 was found in this study. Otherwise, our results suggest that the decreased accumulation of erythritol in theromotolerant cells mainly results from the degradation of erythritol, a process that may be conducive for cells to survive under thermal stress. For one thing, the expression of genes responsible for erythritol degradation was increased (Fig. 6). For another, overexpression of the *A002375* coding for EYI2 enabled *Y. lipolytica* CA20 to grow at 34 °C and reduced the erythritol titer.

Alteration of membrane lipid composition is one important way for cells to resist thermal stress. A previous study revealed that a change in sterol composition, from ergosterol to fecosterol, caused by mutations in the C-5 sterol desaturase gene and an increased expression of genes involved in sterol biosynthesis render *S. cereviae* thermotolerant (Caspeta et al. 2014). Sterols have an important role in the regulation of membrane fluidity. Similarly, we found that abundant genes involved in steroid synthesis were upregulated in thermotolerant *Y. lipolytica*. In addition, it is noteworthy that cells appear to increase ceramide synthesis to adapt to thermal stress since the expression levels of *A003183* and *A004782* related to ceramide synthesis have increased. Furthermore, overexpression of *A003183* enabled *Y. lipolytica* CA20 to grow at 34 °C. Mirroring the role of ceramide acting as a temperature sensor and signaling molecule in response to thermal stress in *S. cereviae* (Xiao et al. 2022), it is thus rational to conclude that increased synthesis of ceramide contributes to the thermotolerance of *Y. lipolytica*.

### Upregulated genes are good candidates for the construction of thermotolerant strain

In spite of ALE, genetic engineering by directly introducing exogenous heat-resistant devices is another way to obtain thermotolerant erythritol-producing *Y. lipolytica*. For example, Wang and co-authors used a gene *RSP5* encoding ubiquitin ligase from *S. cerevisiae* to improve the thermotolerance of *Yarrowia lipolytica* CGMCC7326, after which the strain could grow at 35 °C and retain an efficient erythritol production capacity at 33 °C (Wang et al. 2020). In another study, heat-resistant devices, including stress response proteins, heat shock proteins, and ubiquitin ligases, from thermophiles were introduced into *Y. lipolytica* to create strains capable of growing at 35 °C (Liang et al. 2023b). Although an introduction of exogenous genes could endow strains with a thermotolerant phenotype, sometimes, overexpression of these genes may produce an unknown effect, thereby influencing their industrial performance. In comparison with exogenous genes, the use of endogenous genes to engineer thermotolerant strains may be an alternative choice in some cases. Qiu and co-authors verified that the thiamine pathway contributed substantially to the thermotolerance of *Y. lipolytica* BBE-18, while an overexpression of three genes related to thiamine synthesis in BBE-17 elevated its growth temperature from 30 to 33 °C (Qiu et al. 2021). Similarly, overexpression of the heat shock protein Hsp90 improved the growth temperature of* Y. lipolytica* CGMCC7326 to 35 °C (Zhang et al. 2022). Nevertheless, at present, endogenous genes suitable for the construction of thermotolerant strains are inadequate. Our results suggest that genes with a high expression level are good candidates for the construction of thermotolerant strains. For one thing, overexpression of 11 out of 18 genes alone enabled CA20 to grow at 34 °C, of which *A000121*, *A003183*, and *A005690* had the best effect. For another, overexpression of these genes also preserved erythritol production at elevated temperatures. Nevertheless, strains with an individual overexpression of these genes were not able to grow at temperatures higher than 34 °C, suggesting that the thermotolerant phenotype obtained by evolution cannot be simply achieved by overexpressing an individual gene. Possibly, integrative expression of these genes could endow the parental strain with a better thermotolerant phenotype than individual expression, which needs future investigation. In addition, it should be pointed out that most of these genes encode hypothetical proteins; their detailed functions also need further study. Finally, ALE integrated with an overexpression of these genes would be a promising way to further shorten the time needed for the breeding of thermotolerant strains.

In this study, we obtained thermotolerant erythritol-producing *Y. lipolytica* strains and investigated their thermotolerance mechanisms at genomic and transcriptional levels, through which some novel insights were revealed. First, ALE together with mutagenesis could shorten the breeding period of thermotolerant erythritol-producing *Y. lipolytica*. Second, at a genomic level, cells tune the expression of genes by mutating the global regulators instead of the genes involved in specific metabolic pathways to adapt to thermal stress. Third, cells strengthen genome stability by increasing the expression of genes involved in DNA replication and repair and the degradation of BCAA, FFA, and erythritol to ensure cell proliferation upon thermal stress. Fourth, cells alter the membrane lipid compositions by increasing the synthesis of ceramide and steroid to tolerate thermal stress. Finally, upregulated genes identified by transcriptome analysis are good candidates for the construction of thermotolerant strains. Our findings will benefit the future breeding of thermotolerant erythritol-producing *Y. lipolytica* and the study of thermotolerance mechanisms in *Y. lipolytica*.

## Supplementary Information

Below is the link to the electronic supplementary material.

Supplementary file1 (PDF 1007 KB)

## Data Availability

The RNA-seq experiment results have been submitted to the Sequence Read Archive (SRA) under accession number PRJNA1001984. All data generated or analyzed during this study are included in the manuscript and its supplemental material. Further inquiries are available from the corresponding author on reasonable request.

## Declarations

This paper does not contain any studies with human participants or animals.
