# Supplementary material for: Adaptive responses of erythritol-producing Yarrowia lipolytica to thermal stress after evolution
Source: Appl Microbiol Biotechnol. 2024 Mar 15;108:263. doi: 10.1007/s00253-024-13103-8 (PMC10943161; doi:10.1007/s00253-024-13103-8)
Supplement: Supplementary file 1 [file 253_2024_13103_MOESM1_ESM.pdf]

Applied Microbiology and Biotechnology

Adaptive responses of erythritol-producing *Yarrowia lipolytica* to thermal stress after evolution

Kai Xia[1, 2, 3], Yuqing Chen[1], Fangmei Liu[1], Xuequn Zhao[2], Ruyi Sha[1, 2, 3], Jun Huang[1, 2, 3*]

[1]School of Biological and Chemical Engineering, Zhejiang University of Science and Technology, Hangzhou 310023, China

[2]Key Laboratory of Chemical and Biological Processing Technology for Farm Products of Zhejiang Province, Zhejiang University of Science and Technology, Hangzhou 310023, China

[3]Zhejiang Provincial Collaborative Innovation Center of Agricultural Biological Resources Biochemical Manufacturing, Zhejiang University of Science and Technology, Hangzhou 310023, China

[*] Address correspondence to Jun Huang, huangjun@zust.edu.cn

Table S1 The strains and plasmids used in this study

Strains or plasmids	Descriptions or characteristics [a]	Source
Strains		
Yarrowia lipolytica CA20	An erythritol high-producing strain that was used for the breeding of thermotolerant strains, and this strain was grown at 30 °C	Liu et al. 2023
Yarrowia lipolytica HT34	A thermotolerant strain capable of growing at 34 °C that was derived from CA20	This study
Yarrowia lipolytica HT36	A thermotolerant strain capable of growing at 36 °C that was derived from CA20	This study
Yarrowia lipolytica HT385	A thermotolerant strain capable of growing at 38.5 °C that was derived from CA20	This study
Yarrowia lipolytica HT34R	HT34 was grown at 30 °C for 30 days, after which it was re-grown at 34 °C	This study
Yarrowia lipolytica HT36R	HT36 was grown at 30 °C for 30 days, after which it was re-grown at 36 °C	This study
Yarrowia lipolytica HT385R	HT3385 was grown at 30 °C for 30 days, after which it was re-grown at 38.5 °C	This study
Yarrowia lipolytica CA20Δ*ura3*	The gene *ura3* coding for orotidine-5'-phosphate decarboxylase was deleted in CA20	This study
Yarrowia lipolytica CA20Δ*ura3*::pDCXRA	The strain CA20Δ*ura3* containing the expression vector pDCXRA.	This study
Yarrowia lipolytica CA20Δ*ura3*::*A000121*	The strain CA20Δ*ura3* with the overexpression of gene *A000121* located in the plasmid pDCXRA121	This study
Yarrowia lipolytica CA20Δ*ura3*::*A000184*	The strain CA20Δ*ura3* with the overexpression of gene *A000184* located in the plasmid pDCXRA184	This study
Yarrowia lipolytica CA20Δ*ura3*::*A000800*	The strain CA20Δ*ura3* with the overexpression of gene *A000800* located in the plasmid pDCXRA800	This study
Yarrowia lipolytica CA20Δ*ura3*::*A001678*	The strain CA20Δ*ura3* with the overexpression of gene *A001678* located in the plasmid pDCXRA1678	This study
Yarrowia lipolytica CA20Δ*ura3*::*A002175*	The strain CA20Δ*ura3* with the overexpression of gene *A002175* located in the plasmid pDCXRA2175	This study
Yarrowia lipolytica CA20Δ*ura3*::*A002375*	The strain CA20Δ*ura3* with the overexpression of gene *A002375* located in the plasmid pDCXRA2375	This study
Yarrowia lipolytica CA20Δ*ura3*::*A002808*	The strain CA20Δ*ura3* with the overexpression of gene *A002808* located in the plasmid pDCXRA2808	This study
Yarrowia lipolytica CA20Δ*ura3*::*A003183*	The strain CA20Δ*ura3* with the overexpression of gene *A003183* located in the plasmid pDCXRA3183	This study
Yarrowia lipolytica CA20Δ*ura3*::*A003902*	The strain CA20Δ*ura3* with the overexpression of gene *A003902* located in the plasmid pDCXRA3902	This study
Yarrowia lipolytica CA20Δ*ura3*::*A004055*	The strain CA20Δ*ura3* with the overexpression of gene *A004055* located in the plasmid pDCXRA4055	This study
Yarrowia lipolytica CA20Δ*ura3*::*A004467*	The strain CA20Δ*ura3* with the overexpression of gene *A004467* located in the plasmid pDCXRA4467	This study
Yarrowia lipolytica CA20Δ*ura3*::*A004535*	The strain CA20Δ*ura3* with the overexpression of gene *A004535* located in the plasmid pDCXRA4535	This study
Yarrowia lipolytica CA20Δ*ura3*::*A004625*	The strain CA20Δ*ura3* with the overexpression of gene *A004625* located in the plasmid	This study

	pDCXRA4625	
Yarrowia lipolytica CA20Δ*ura3*::*A004733*	The strain CA20Δ*ura3* with the overexpression of gene *A004733* located in the plasmid pDCXRA4733	This study
Yarrowia lipolytica CA20Δ*ura3*::*A005690*	The strain CA20Δ*ura3* with the overexpression of gene *A005690* located in the plasmid pDCXRA5690	This study
Yarrowia lipolytica CA20Δ*ura3*::*A005844*	The strain CA20Δ*ura3* with the overexpression of gene *A005844* located in the plasmid pDCXRA5844	This study
Yarrowia lipolytica CA20Δ*ura3*::*A006220*	The strain CA20Δ*ura3* with the overexpression of gene *A006220* located in the plasmid pDCXRA6220	This study
Yarrowia lipolytica CA20Δ*ura3*::*A006279*	The strain CA20Δ*ura3* with the overexpression of gene *A006279* located in the plasmid pDCXRA6279	This study
Escherichia coli DH5α	This strain was used for cloning and plasmid maintenance	TaKaRa Bio Inc. (Changping, Beijing, China)
Plasmids		
pHSG398	*E. coli* cloning vector. This plasmid cannot replicate in *Y. lipolytica*, and was used for the construction of plasmids used in *ura3* disruption. Cm^R	TaKaRa Bio Inc. (Changping, Beijing, China)
pHU	*E. coli* cloning vector. The upstream homologous fragment of *ura3* (1 kb) was inserted into the plasmid pHSG398. Cm^R	This study
pHUD	*E. coli* cloning vector. The downstream homologous fragment of *ura3* (1 kb) was inserted into the plasmid pHU. Cm^R	This study
pHUDR	*E. coli* cloning vector. The *ura3* expression cassette using TEF1 and CYC1 as the promoter and terminator respectively were inserted into the plasmid pHUD. Cm^R	This study
pCRISPRyl	Yeast expression vector. Amp^R	Addgene (#70007)
pDCX5	Yeast expression vector. The gene coding for Cas9 was deleted. Amp^R	This study
pDCX10	Yeast expression vector. The *ura3* expression cassette was inserted into the pDCX5	This study
pDCXRA	Yeast expression vector. The autonomously replicating sequence ARS1 from CA20 chromosome was cloned and inserted into the plasmid pDCXRA	This study
pDCXRA121	Plasmid pDCXRA containing the gene sequence of *A000121* with the promoter UAS1B8-TEF and the terminator CYC1, respectively	This study
pDCXRA184	Plasmid pDCXRA containing the gene sequence of *A000184* with the promoter UAS1B8-TEF and the terminator CYC1, respectively	This study
pDCXRA800	Plasmid pDCXRA containing the gene sequence of *A000800* with the promoter UAS1B8-TEF and the terminator CYC1, respectively	This study
pDCXRA1678	Plasmid pDCXRA containing the gene sequence of *A001678* with the promoter UAS1B8-TEF and the terminator CYC1, respectively	This study
pDCXRA2175	Plasmid pDCXRA containing the gene sequence of *A002175* with the promoter UAS1B8-TEF and the terminator CYC1, respectively	This study
pDCXRA2375	Plasmid pDCXRA containing the gene sequence of *A002375* with the promoter UAS1B8-TEF and the terminator CYC1, respectively	This study

pDCXRA2808	Plasmid pDCXRA containing the gene sequence of *A002808* with the promoter UAS1B8-TEF and the terminator CYC1, respectively	This study
pDCXRA3183	Plasmid pDCXRA containing the gene sequence of *A003183* with the promoter UAS1B8-TEF and the terminator CYC1, respectively	This study
pDCXRA3902	Plasmid pDCXRA containing the gene sequence of *A003902* with the promoter UAS1B8-TEF and the terminator CYC1, respectively	This study
pDCXRA4055	Plasmid pDCXRA containing the gene sequence of *A004055* with the promoter UAS1B8-TEF and the terminator CYC1, respectively	This study
pDCXRA4467	Plasmid pDCXRA containing the gene sequence of *A004467* with the promoter UAS1B8-TEF and the terminator CYC1, respectively	This study
pDCXRA4535	Plasmid pDCXRA containing the gene sequence of *A004535* with the promoter UAS1B8-TEF and the terminator CYC1, respectively	This study
pDCXRA4625	Plasmid pDCXRA containing the gene sequence of *A004625* with the promoter UAS1B8-TEF and the terminator CYC1, respectively	This study
pDCXRA4733	Plasmid pDCXRA containing the gene sequence of *A004733* with the promoter UAS1B8-TEF and the terminator CYC1, respectively	This study
pDCXRA5690	Plasmid pDCXRA containing the gene sequence of *A005690* with the promoter UAS1B8-TEF and the terminator CYC1, respectively	This study
pDCXRA5844	Plasmid pDCXRA containing the gene sequence of *A005844* with the promoter UAS1B8-TEF and the terminator CYC1, respectively	This study
pDCXRA6220	Plasmid pDCXRA containing the gene sequence of *A006220* with the promoter UAS1B8-TEF and the terminator CYC1, respectively	This study
pDCXRA6279	Plasmid pDCXRA containing the gene sequence of *A006279* with the promoter UAS1B8-TEF and the terminator CYC1, respectively	This study

[a] Cm^R chloramphenicol resistance, Amp^R ampicillin resistance

Table S2 Primers used in this study

Name	Sequence (5'-3')	Function
M13R	CAGGAAACAGCTATGAC	These primers were used for the verification of the DNA fragments inserted into the multiple cloning site (MCS) of pHSG398
M13F	GTAAAACGACGGCCAGT	
PU-F	GACCATGATTACGAATTCaaagcaattgttaacaagatac	These primers were used for the cloning of the 1, 000-bp upstream sequence of *ura3* from the genome DNA of CA20
UD-R	CATATCCATAGTCTAACCTtttggtggtgaagaggagac	
UD-F	gtctcctcttcaccaccaaaAGGTTAGACTATGGATATG	These primers were used for the cloning of the 1, 000-bp downstream sequence of *ura3* from the genome DNA of CA20
DH-R	cgacggccagtgccaagcttCATCTCTACTACCGAGCCTAC	
DH-F	GTAGGCTCGGTAGTAGAGATGaagcttggcactggccgtcg	These primers were used for the cloning of the pHSG398
PU-R	gtatcttgttaacaattgctttGAATTCGTAATCATGGTC	
V5-F	CGACAACAATATCAGCTGC	These primers were used for the verification of the deletion of *ura3*
V5-R	CAGATCATGCGGATACACA	
121-F	GTGACATAACTAATTACATGAtcacacaactctctcaacc	These primers were used for the amplification of gene *A000121* from CA20 genome
121-R	GTATAAGAATCATTCAAAGatggactggaaactacagatc	
P121-F	gatctgtagtttccagtccatCTTTGAATGATTCTTATAC	These primers were used for the linearization of the plasmid pDCXRA, which was used for the construction of pDCXRA121
P121-R	ggttgagagagttgtgtgaTCATGTAATTAGTTATGTCAC	
184-F	GACATAACTAATTACATGAttacaacttcgactgggaacc	These primers were used for the amplification of gene *A000184* from CA20 genome
184-R	GAGTATAAGAATCATTCAAAGatgaccgactccctctcgctc	
P184-F	gagcgagagggagtcggtcatCTTTGAATGATTCTTATACTC	These primers were used for the linearization of the plasmid pDCXRA, which was used for the construction of pDCXRA184
P184-R	ggttcccagtcgaagttgtaaTCATGTAATTAGTTATGTC	
800-F	GACATAACTAATTACATGAttacatgaggaaaacaatcac	These primers were used for the amplification of gene *A000800* from CA20 genome
800-R	GAGTATAAGAATCATTCAAAGatgcgtttcacaactctcac	
P800-F	gtgagagttgtgaaacgcatCTTTGAATGATTCTTATACTC	These primers were used for the linearization of the plasmid pDCXRA, which was used for the construction of pDCXRA800
P800-R	gtgattgttttcctcatgtaaTCATGTAATTAGTTATGTC	

1678-F	GACATAACTAATTACATGAtcaaatggatccagaaacag	These primers were used for the amplification of gene *A001678*
1678-R	GTATAAGAATCATTCAAAGatgcttctctcaaacacc	from CA20 genome
P1678-F	ggtgtttgagagaagcatCTTTGAATGATTCTTATAC	These primers were used for the linearization of the plasmid
P1678-R	ctgtttctggatccatttgaTCATGTAATTAGTTATGTC	pDCXRA, which was used for the construction of pDCXRA1678
2175-F	GACATAACTAATTACATGActagtccttggcagcaacaatg	These primers were used for the amplification of gene *A002175*
2175-R	GTATAAGAATCATTCAAAGatgctcttcaaaactcttctc	from CA20 genome
P2175-F	gagaagagttttgaagagcatCTTTGAATGATTCTTATAC	These primers were used for the linearization of the plasmid
P2175-R	cattgttgctgccaaggactagTCATGTAATTAGTTATGTC	pDCXRA, which was used for the construction of pDCXRA2175
2375-F	GACATAACTAATTACATGAttaggcagcaatgtgcttc	These primers were used for the amplification of gene *A002375*
2375-R	GTATAAGAATCATTCAAAGatgcggcggctttccctttg	from CA20 genome
P2375-F	caaaagggaaagccgccgcatCTTTGAATGATTCTTATAC	These primers were used for the linearization of the plasmid
P2375-R	gaagcacattgctgcctaaTCATGTAATTAGTTATGTC	pDCXRA, which was used for the construction of pDCXRA2375
2808-F	GACATAACTAATTACATGActagagcttactaagtttaac	These primers were used for the amplification of gene *A002808*
2808-R	GTATAAGAATCATTCAAAGatgatcatccacacgtccg	from CA20 genome
P2808-F	cggacgtgtggatgatcatCTTTGAATGATTCTTATAC	These primers were used for the linearization of the plasmid
P2808-R	gttaaacttagtaagctctagTCATGTAATTAGTTATGTC	pDCXRA, which was used for the construction of pDCXRA2808
3183-F	GACATAACTAATTACATGActatttcttctcgccccatg	These primers were used for the amplification of gene *A003183*
3183-R	GAGTATAAGAATCATTCAAAGatgccccactcacagaaac	from CA20
P3183-F	gtttctgtgagtggggcatCTTTGAATGATTCTTATACTC	These primers were used for the linearization of the plasmid
P3183-R	catggggcgagaagaaatagTCATGTAATTAGTTATGTC	pDCXRA, which was used for the construction of pDCXRA3183
3902-F	GACATAACTAATTACATGActatctcaagttagccaatg	These primers were used for the amplification of gene *A003902*
3902-R	GTATAAGAATCATTCAAAGatgaactccggataccattac	from CA20
P3902-F	gtaatggtatccggagttcatCTTTGAATGATTCTTATAC	These primers were used for the linearization of the plasmid
P3902-R	cattggctaacttgagatagTCATGTAATTAGTTATGTC	pDCXRA, which was used for the construction of pDCXRA3902
4055-F	GACATAACTAATTACATGActaatgtccttcgaagtccgg	These primers were used for the amplification of gene *A004055*

4055-R	GTATAAGAATCATTCAAAGatgaacttcgactatgactttc	from CA20
P4055-F	gaaagtcatagtcgaagttcatCTTTGAATGATTCTTATAC	These primers were used for the linearization of the plasmid
P4055-R	ccggacttcgaaggacattagTCATGTAATTAGTTATGTC	pDCXRA, which was used for the construction of pDCXRA4055
4467-F	GACATAACTAATTACATGAttaggcatgggcaccgggcatc	These primers were used for the amplification of gene *A004467*
4467-R	GTATAAGAATCATTCAAAGatgtcccagccaatcgatc	from CA20 genome
P4467-F	gatcgattggctgggacatCTTTGAATGATTCTTATAC	These primers were used for the linearization of the plasmid
P4467-R	gatgcccggtgcccatgcctaaTCATGTAATTAGTTATGTC	pDCXRA, which was used for the construction of pDCXRA4467
4535-F	GACATAACTAATTACATGAtcaatggtatttctggctgatg	These primers were used for the amplification of gene *A004535*
4535-R	GACATAACTAATTACATGAtcaatggtatttctggctgatg	from CA20 genome
P4535-F	cccaagagtatccgtacatCTTTGAATGATTCTTATAC	These primers were used for the linearization of the plasmid
P4535-R	catcagccagaaataccattgaTCATGTAATTAGTTATGTC	pDCXRA, which was used for the construction of pDCXRA4535
4625-F	GACATAACTAATTACATGAttagaaaagcatggcggcag	These primers were used for the amplification of gene *A004625*
4625-R	GTATAAGAATCATTCAAAGatgcacttctccgccgtcc	from CA20 genome
P4625-F	ggacggcggagaagtgcatCTTTGAATGATTCTTATAC	These primers were used for the linearization of the plasmid
P4625-R	ctgccgccatgcttttctaaTCATGTAATTAGTTATGTC	pDCXRA, which was used for the construction of pDCXRA4625
4733-F	GACATAACTAATTACATGActacactttgctatcatcac	These primers were used for the amplification of gene *A004733*
4733-R	GTATAAGAATCATTCAAAGatgagcaccaattcgactctg	from CA20 genome
P4733-F	cagagtcgaattggtgctcatCTTTGAATGATTCTTATAC	These primers were used for the linearization of the plasmid
P4733-R	gtgatgatagcaaagtgtagTCATGTAATTAGTTATGTC	pDCXRA, which was used for the construction of pDCXRA4733
5690-F	GACATAACTAATTACATGAttagtaatccttcttgtgag	These primers were used for the amplification of gene *A005690*
5690-R	GTATAAGAATCATTCAAAGatgtctcacactgttgatc	from CA20 genome
P5690-F	gatcaacagtgtgagacatCTTTGAATGATTCTTATAC	These primers were used for the linearization of the plasmid
P5690-R	ctcacaagaaggattactaaTCATGTAATTAGTTATGTC	pDCXRA, which was used for the construction of pDCXRA5690
5844-F	GACATAACTAATTACATGActactcctgataacccatatc	These primers were used for the amplification of gene *A005844*
5844-R	GTATAAGAATCATTCAAAGatgttcccctaccaccaac	from CA20 genome
P5844-F	gttggtggtaggggaacatCTTTGAATGATTCTTATAC	These primers were used for the linearization of the plasmid

P5844-R	gatatgggttatcaggagtagTCATGTAATTAGTTATGTC	pDCXRA, which was used for the construction of pDCXRA5844
6220-F	GACATAACTAATTACATGAtcaaaatagcagcacaattg	These primers were used for the amplification of gene *A006220*
6220-R	GTATAAGAATCATTCAAAGatgaacacctcatacacagc	from CA20 genome
P6220-F	gctgtgtatgaggtgttcatCTTTGAATGATTCTTATAC	These primers were used for the linearization of the plasmid
P6220-R	caattgtgctgctattttgaTCATGTAATTAGTTATGTC	pDCXRA, which was used for the construction of pDCXRA6220
6279-F	GACATAACTAATTACATGActacactttgctatcatcac	These primers were used for the amplification of gene *A006279*
6279-R	GTATAAGAATCATTCAAAGatggacgcagcgggttgattc	from CA20 genome
P6279-F	gaatcaacccgctgcgtccatCTTTGAATGATTCTTATAC	These primers were used for the linearization of the plasmid
P6279-R	gtgatgatagcaaagtgtagTCATGTAATTAGTTATGTC	pDCXRA, which was used for the construction of pDCXRA6279
CX5-F	GGCTAGCatcgaCTTTGAATGATTCTTATACTCAG	These primers were used for the construction of plasmid pDCX5
CX5-R	tcgatGCTAGCCTCATGTAATTAGTTATGTCACGC	
X5U-F	TAAATCATCATTTCATTAGCCATAGCTTCAAAATGTTTCTAC	These primers were used for the amplification of the *ura3*
X5U-R	GTAGAAACATTTTGAAGCTATGGCTAATGAAATGATGATTTA	expression cassette
UCX-F	GACATAACTAATTACATGAGACGGAGCCAAAAAATGGAG	These primers were used for the amplification of the plasmid
UCX-R	CTCCATTTTTTGGCTCCGTCTCATGTAATTAGTTATGTC	pDCX5
ARS-F	GAAACGCGCGAGACGAAAGGcatactactgtatattcaag	These primers were used for the amplification of the ARS1 from
ARS-R	CGTTTTGGAAACAGTCTTCctcatttacaatttcatctttc	the genome of CA20
PARS-F	gaaagatgaaattgtaaatgagGAAGACTGTTTCCAAAACG	These primers were used for the amplification of the plasmid
PARS-R	cttgaatatacagtagtatgCCTTTCGTCTCGCGCGTTTC	pDCX10

Table S3 The common DEGs found among groups YLX6 vs YLX3, YLX8 vs YLX3, and YLX8 vs YLX6

Gene ID	Protein ID	Log_2FC			
		YLX6 vs YLX3	YLX8 vs YLX3	YLX8 vs YLX6	Product
A000121	SEI35958	9.5	12.0	2.5	YALIA101S09e02762g1_1
A000126	AOW00831	2.3	4.0	1.8	Hypothetical protein YALI1_A18915g
A000145	CAG84113	1.8	5.6	3.8	YALI0A17919p
A000147	RDW30058	1.8	3.9	2.1	Aldehyde/histidinol dehydrogenase
A000353	VBB88725	1.9	4.5	2.5	Uncharacterized MFS-type transporter
A000378	RDW43304	5.3	3.1	-2.2	Hypothetical protein B0I74DRAFT_148865
A000413	AOW00445	3.1	5.2	2.0	Hypothetical protein YALI1_A09190g
A000501	RDW31362	-2.0	4.3	6.3	Amino acid permease/SLC12A domain-containing protein
A000599	SEI31583	1.9	7.3	5.3	YALIA101S01e26786g1_1
A000683	RMI95914	5.1	8.0	2.9	Hypothetical protein BD777DRAFT_162728
A000800	RDW42845	4.3	7.1	2.8	Hypothetical protein B0I74DRAFT_119247
A000881	RDW49650	1.9	-2.4	-4.3	Potassium transporter-domain-containing protein
A000981	VBB85382	2.4	4.3	1.8	Hypothetical protein conserved in the Yarrowia clade
A001010	RDW38004	2.1	-1.5	-3.6	Hypothetical protein B0I73DRAFT_134516
A001030	VBB85434	2.3	5.0	2.7	Dihydrokaempferol 4-reductase, putative
A001085	RDW51663	4.9	2.7	-2.2	Hypothetical protein B0I75DRAFT_165066
A001142	RDW41035	-3.8	-1.5	2.3	Ferric reductase NAD binding domain-domain-containing protein
A001243	RDW30841	2.3	5.0	2.8	Hypothetical protein B0I72DRAFT_140867
A001305	-	-1.5	2.0	3.5	_
A001336	RDW35219	2.2	4.1	1.9	Hypothetical protein B0I72DRAFT_106542
A001380	RDW35266	-1.4	3.0	4.4	Amino acid permease/SLC12A domain-containing protein
A001395	RDW35281	2.2	4.4	2.1	Hypothetical protein B0I72DRAFT_165164
A001499	RDW31526	2.0	4.1	2.1	Hypothetical protein B0I72DRAFT_139688
A001545	RDW33866	2.0	4.1	2.1	Hypothetical protein B0I72DRAFT_135547
A001602	VBB86065	2.2	6.5	4.2	4-Hydroxyphenylpyruvate dioxygenase
A001603	VBB86067	3.5	6.6	3.0	YALIH222S03e30042g1_1
A001667	AOW02120	2.3	5.5	3.2	Hypothetical protein YALI1_B30293g
A001668	RMI94039	2.8	5.8	2.9	Hypothetical protein BD777DRAFT_149925

A001671	VBB89490	3.7	2.3	-1.5	Conserved hypothetical protein
A001672	VBB89491	2.0	3.9	1.8	Hypothetical protein conserved in the *Yarrowia* clade
A001678	SEI37011	3.4	5.6	2.2	YALIA101S17e00276g1_1
A001981	CAG82183	-2.1	-4.7	-2.6	YALI0C15466p
A001982	VBB89072	2.0	5.9	3.9	Monocarboxylate/proton symporter of the plasma membrane, putative
A001986	RDW49919	4.0	6.3	2.3	Hypothetical protein B0I75DRAFT_167879
A001993	AOW02948	2.3	5.1	2.8	Hypothetical protein YALI1_C22944g
A001998	VBB89312	2.0	4.0	1.9	Hypothetical protein conserved in the *Yarrowia* clade
A002040	CAG82256	-1.6	-4.6	-3.0	YALI0C17237p
A002096	VBB89214	3.8	6.2	2.4	Hypothetical protein conserved in the *Yarrowia* clade
A002125	RDW24184	4.0	-3.8	-7.8	Hypothetical protein B0I71DRAFT_122374
A002138	VBB89166	7.2	3.0	-4.2	3-Methylbutanal reductase and NADPH-dependent methylglyoxal reductase
A002174	RDW53418	3.3	5.4	2.1	Hypothetical protein B0I75DRAFT_81652
A002175	RDW32666	4.4	6.2	1.8	Hypothetical protein B0I72DRAFT_164575
A002210	RDW29663	2.7	4.7	2.0	HotDog domain-containing protein
A002359	RDW33943	-1.3	-4.7	-3.3	Aquaporin-like protein
A002375	RDW42622	5.7	7.8	2.1	Hypothetical protein B0I73DRAFT_91619, partial
A002390	RDW35034	2.3	4.5	2.2	Glycoside hydrolase superfamily
A002467	VBB83368	4.2	6.8	2.6	Hypothetical protein YALIH222_S02E30240G
A002600	-	5.2	7.6	2.4	_
A002617	RDW55235	2.6	5.0	2.4	Hypothetical protein B0I75DRAFT_171555
A002808	RDW50623	5.4	8.1	2.7	Hypothetical protein B0I75DRAFT_167065
A002863	-	3.6	1.9	-1.7	_
A002899	RDW41822	-2.7	-4.1	-1.4	POT family-domain-containing protein
A002928	RDW32947	3.1	5.1	2.0	VIT family-domain-containing protein
A003021	AOW04480	3.6	6.5	2.9	Hypothetical protein YALI1_D29020g
A003071	SEI35179	2.3	4.3	2.0	YALIA101S06e03862g1_1
A003104	SEI35147	1.9	3.9	2.0	YALIA101S06e03092g1_1
A003163	RDW31092	2.2	5.0	2.8	Aldehyde dehydrogenase domain-containing protein
A003165	VBB88558	-4.9	-2.7	2.2	Conserved hypothetical protein
A003230	VBB88623	1.8	5.1	3.3	YALIH222S04e31582g1_1

A003235	RDW36291	3.1	4.9	1.8	Major facilitator superfamily domain-containing protein
A003272	RDW34726	-4.0	-1.8	2.2	Catalase-like domain-containing protein
A003310	SEI36910	2.6	4.5	1.9	YALIA101S15e00870g1_1
A003343	CAG83972	2.1	4.5	2.5	YALI0A13607p
A003374	RMI95120	3.2	5.0	1.8	Hypothetical protein BD777DRAFT_92003
A003717	VBB78982	2.9	4.7	1.8	Conserved hypothetical protein
A003895	RDW33857	2.2	4.2	1.9	Acyl-CoA dehydrogenase/oxidase
A003902	RDW33865	4.7	7.6	2.9	Hypothetical protein B0I72DRAFT_163816
A004055	RDW34144	2.4	4.8	2.3	Cell wall transcription factor ACE2
A004072	VBB78542	5.5	7.4	1.9	Hypothetical protein conserved in the *Yarrowia* clade
A004096	RMI96456	2.0	4.1	2.1	Glutathione reductase
A004103	RDW33245	3.2	5.4	2.2	Hypothetical protein B0I72DRAFT_110946
A004125	RMI96487	2.0	4.3	2.3	Hypothetical protein BD777DRAFT_162500
A004273	VBB78136	1.9	3.8	1.9	Phospholipase B (lysophospholipase) involved in lipid metabolism
A004396	VBB77897	3.0	6.0	3.0	Hypothetical protein of a 11-member gene family
A004420	AOW05979	-1.8	3.0	4.8	Hypothetical protein YALI1_E31005g
A004467	RDW43099	3.0	5.1	2.1	GPR1/FUN34/yaaH family-domain-containing protein
A004474	KAE8175231	2.7	4.9	2.2	Major facilitator superfamily domain-containing protein
A004479	RDW32159	2.6	4.4	1.8	Acyl-CoA oxidase 4
A004513	RDW34442	3.3	5.1	1.8	POT family-domain-containing protein
A004529	RMI98777	4.2	2.7	-1.6	Hypothetical protein BD777DRAFT_125008
A004535	RDW51119	2.0	7.8	5.8	Hypothetical protein B0I75DRAFT_107230
A004567	RDW34389	2.9	5.6	2.8	Hypothetical protein B0I72DRAFT_134721
A004625	RDW34268	4.5	7.4	2.9	Hypothetical protein B0I72DRAFT_19645
A004755	RDW41314	-1.2	2.4	3.6	Hypothetical protein B0I73DRAFT_167532
A004837	RDW31113	2.0	3.7	1.7	Transporter-domain-containing protein
A004838	VBB89325	3.4	5.5	2.1	Hypothetical protein conserved in the *Yarrowia* clade
A004852	VBB89339	-3.8	-1.4	2.4	Hypothetical protein conserved in the *Yarrowia* clade
A004861	RDW49775	2.2	4.2	2.0	ClpP/crotonase-like domain-containing protein
A004880	RDW40045	-3.6	2.8	6.3	General substrate transporter
A004979	VBB87635	-6.8	-4.8	2.0	Hypothetical protein conserved in the *Yarrowia* clade

A004995	VBB87652	4.3	2.4	-1.9	Conserved hypothetical protein
A005014	VBB87671	2.6	5.5	2.8	Hypothetical protein conserved in the *Yarrowia* clade
A005022	RDW33706	-1.3	3.2	4.4	Glucanosyltransferase-domain-containing protein
A005072	RDW52951	1.8	5.3	3.4	CAP domain-containing protein
A005124	VBB87781	-8.7	-5.5	3.2	High-affinity iron permease
A005184	RDW31504	2.2	4.2	2.0	Thiamine diphosphate-binding protein
A005365	VBB83179	8.2	6.1	-2.1	Conserved hypothetical protein
A005502	RDW41271	4.3	2.3	-2.0	Hypothetical protein B0I73DRAFT_129072
A005659	RDW30593	-8.7	-6.4	2.3	ZIP zinc transporter-domain-containing protein
A005669	RDW31327	2.0	4.3	2.3	Hypothetical protein B0I72DRAFT_140046
A005679	VBB82851	4.2	2.4	-1.8	Hypothetical protein conserved in the *Yarrowia* clade
A005690	RDW31305	4.7	6.8	2.0	GPR1/FUN34/yaaH family-domain-containing protein
A005693	RDW37634	4.3	6.2	1.9	Spo11/DNA topoisomerase VI subunit A
A005798	-	4.2	2.7	-1.5	_
A005844	VBB82684	2.0	4.7	2.8	Conserved hypothetical protein
A005898	RDW42282	3.0	5.7	2.7	Major facilitator superfamily domain-containing protein
A005910	RDW42295	2.2	4.5	2.3	Hypothetical protein B0I73DRAFT_150920
A005940	RDW35888	2.5	4.4	2.0	ClpP/crotonase-like domain-containing protein
A005943	RDW35885	-4.4	-1.2	3.2	Hypothetical protein B0I72DRAFT_132148
A005980	VBB82542	2.5	4.3	1.8	Hypothetical protein conserved in the *Yarrowia* clade
A006066	RDW35599	1.7	4.2	2.5	Hypothetical protein B0I72DRAFT_132801
A006101	VBB82418	1.9	-1.8	-3.6	Cu-binding metallothionein
A006130	VBB82386	2.2	4.5	2.3	Conserved hypothetical protein
A006161	CAG78771	-1.6	-3.9	-2.3	YALI0F27709p
A006220	-	3.4	8.2	4.8	_
A006257	RMI99836	5.1	2.6	-2.4	Hypothetical protein BD777DRAFT_122364

Table S4 Detailed information of the DEGs presented in Fig. 5 and Fig. 6

Gene name	Protein ID	Product	YLX6 vs YLX3 (Log_2FC)	YLX8 vs YLX3 (Log_2FC)
**Signal pathways**				
A001014	VBB85415	Conserved hypothetical protein (Ras GTPase activating domain)	1.74	-
A005979	RDW30516	Ras family-domain-containing protein	-	2.25
**Lipid metabolism**				
A000926	RDW33684	Cytochrome P450	2.21	-
A000927	RDW33684	Cytochrome P450	2.19	-
A000928	RDW33684	Cytochrome P450	3.14	-
A001412	CAG83259	YALI0B17204p	3.74	4.16
A002374	CAG77670	YALI0F01606p	3.78	5.12
A003183	SEI35064	YALIA101S06e01156g1_1	2.60	3.49
A003411	RDW31405	Alpha/Beta hydrolase protein	2.61	2.47
A003797	RDW55913	Hypothetical protein B0I75DRAFT_155626	2.61	
A004119	RDW33227	Peroxisomal 3-oxoacyl-CoA thiolase	1.86	-
A004202	VBB78276	Dihydroxyacetone kinase	2.77	4.49
A004782	RDW32315	Metallo-dependent phosphatase-like protein	1.93	2.98
A004839	RDW31116	Thiolase-like protein	2.08	3.05
**Fatty acid degradation**				
A002282	RDW31980	Acyl-CoA oxidase 5	-	2.06
A003667	RDW35334	Acyl-CoA oxidase-domain-containing protein	1.80	2.95
A004479	RDW32159	Acyl-CoA oxidase 4	2.62	4.44
A004709	5Y9D_B	Acyl-coenzyme A oxidase 1	-	4.32
**Glycolysis and tricarboxylic acid cycle**				
A001750	RDW31054	Pyridoxal phosphate-dependent transferase	-1.37	-
A001768	VBB89592	Tetrameric phosphoglycerate mutase	-1.23	-
A002777	RDW38852	Malate dehydrogenase	-1.21	-
A003610	RDW32985	Isocitrate dehydrogenase	-1.84	-1.66
A004415	RDW31219	Fructose-1,6-bisphosphate (FBP) aldolases	-1.72	-
A004455	VBB77790	Pyruvate Dehydrogenase Beta subunit	-1.50	-
A005245	RDW30678	TPP-dependent 2-oxoacid decarboxylase	-1.94	-1.45
A005351	RMI97733	Phosphoglucose isomerase	-1.18	-
A005412	RDW33187	Pyruvate kinase	-1.51	-
A005710	RDW33771	Enolase	-1.97	
**Amino acid metabolism and propanoate metabolism**				
A001092	RDW40301	Aldehyde/histidinol dehydrogenase	-2.39	-
A001367	CAG83217	YALI0B16214p	-	3.12
A002420	VBB83416	3-Hydroxyisobutyrate dehydrogenase	3.38	4.40

A002522	VBB83312	3-Methyl-2-oxobutanoate dehydrogenase (Lipoamide) E1 beta chain	3.05	4.27
A002654	RDW32208	Type-1 glutamine synthetase 2	-	-1.03
A003071	SEI35179	YALIA101S06e03862g1_1	2.31	4.34
A003895	RDW33857	Acyl-CoA dehydrogenase/oxidase	2.23	4.16
A003896	VBB78791	Methylcrotonoyl-CoA carboxylase	2.74	3.96
A004839	RDW31116	Thiolase-like protein	2.08	3.05
A005184	RDW31504	Thiamine diphosphate-binding protein	2.19	4.16
A005380	RMI97763	Pyridoxal phosphate-dependent transferase	-2.96	-2.93
A005474	SEI32258	YALIA101S02e13256g1_1	2.62	-
A005754	RDW31247	Glutamate/Leucine/Phenylalanine/Valine Dehydrogenase-domain-containing protein	-	-1.08
A005940	RDW35888	ClpP/crotonase-like domain-containing protein	2.46	4.27
A006115	RDW35546	Probable succinyl-CoA:3-ketoacid coenzyme A transferase	1.86	3.13
A006263	RDW34082	FAD dependent oxidoreductase	-	1.86
**Oxidative stress response**				
A000184	RDW35377	Alpha/Beta hydrolase protein	3.40	4.26
A000525	CAG81606	Peroxisome biogenesis factor 10 (Q00940)	1.81	-
A002165	RDW32656	Redoxin	-2.80	-1.29
A002252	VBB89755	Deoxycytidine monophosphate (dCMP) deaminase	1.98	2.56
A002350	RDW33954	Peroxisomal biogenesis factor 2	-	1.98
A002638	RDW32192	FMN-dependent dehydrogenase	-	3.03
A003191	RDW34343	Peroxisome assembly protein 12	1.72	-
A003253	RDW34706	Catalase-like domain-containing protein	-2.57	-
A003272	RDW34726	Catalase-like domain-containing protein	-3.98	-1.77
A003667	RDW35334	Acyl-CoA oxidase	1.80	2.95
A003958	RDW35810	Peroxisomal 2,4-dienoyl-CoA reductase SPS19	-	2.38
A004119	RDW33227	Peroxisomal 3-oxoacyl-CoA thiolase	1.86	-
A004170	RDW29554	Alpha/Beta hydrolase protein	1.96	-
A005374	RDW34952	NUDIX hydrolase domain-like protein	2.66	-
A005901	RDW35929	Choline/Carnitine o-acyltransferase-domain-containing protein	-	1.94
A006263	RDW34082	FAD dependent oxidoreductase	-	1.86
A006279	VBB82226	Catalase	2.25	3.80
**Ubiquitin mediated proteolysis**				
A000608	RDW29970	WD40-repeat-containing domain protein	-	3.31
A001803	RDW34530	Ubiquitin-conjugating enzyme E2S	-	1.91

A002553	RDW32545	Ubiquitin-conjugating enzyme/RWD-like protein	-	1.86
**DNA replication, repair, and homologous recombination**				
A000180	RDW35373	MCM2/3/5 family-domain-containing protein	1.82	-
A000201	RDW35400	P-loop containing nucleoside triphosphate hydrolase protein	1.86	-
A000385	VBB88692	Catalytic subunit of DNA polymerase delta, putative	1.89	2.10
A001013	CAG82840	DNA repair protein	3.30	2.51
A001165	RDW35185	DNA primase small subunit	1.78	-
A001226	RDW55429	DNA glycosylase	-1.39	-
A001477	RDW31563	MCM2/3/5 family-domain-containing protein	-	1.90
A001588	VBB86035	Conserved hypothetical protein	-	-1.62
A002035	VBB89276	Poly [ADP-ribose] polymerase 2	-	3.12
A002480	VBB83356	Conserved hypothetical protein	2.59	-
A003364	VBB89698	Ribonuclease H	-	-1.61
A004005	RDW31004	Replication factor A protein 1	1.97	-
A004466	RDW30371	P-loop containing nucleoside triphosphate hydrolase protein	-	1.93
A004732	VBB77462	Tripartite DNA replication factor	2.28	2.33
A004982	VBB87638	Serine/threonine-protein kinase TEL1	-1.27	-
A004995	VBB87652	Uracil-DNA glycosylase	4.25	2.36
A005285	RDW33374	Replication factor A protein 3	2.72	-
A005661	RDW30595	Rad51-domain-containing protein	2.74	-
A006112	RMI99986	DNA mismatch repair protein	-	1.93
A006185	RDW29784	DNA polymerase epsilon subunit B	2.34	-
**Transporter system**				
A000353	VBB88725	Uncharacterized MFS-type transporter	1.9	4.5
A000501	RDW31362	Amino acid permease/SLC12A domain-containing protein	-2.0	4.3
A000729	RDW29515	General substrate transporter	-	3.88
A000826	VBB85220	Plasma membrane low glucose sensor	3.53	5.07
A000881	RDW49650	Potassium transporter-domain-containing protein	1.9	-2.4
A000973	SEI31194	YALIA101S01e17150g1_1	-	2.19
A001982	VBB89072	Monocarboxylate/proton symporter of the plasma membrane	2.0	5.9
A002359	RDW33943	Aquaporin-like protein	-4.7	-4.3
A003235	RDW36291	Major facilitator superfamily domain-containing protein	4.9	1.8
A004474	KAE8175231	Major facilitator superfamily domain-containing	2.7	4.9

		protein		
A004837	RDW31113	Major facilitator superfamily domain-containing protein	2.0	3.7
A005124	VBB87781	High-affinity iron permease	-8.7	-5.5
A005659	RDW30593	ZIP zinc transporter-domain-containing protein	-8.7	-6.4
A005898	RDW42282	Major facilitator superfamily domain-containing protein	3.0	5.7
**Erythritol metabolism**				
A001342 (GND1)	CAG83189	Phosphogluconate dehydrogenase	-2.10	-
A001913 (ER25)	RDW38703	Erythrose reductase	-	2.31
A002373 (EYI1)	VBB83462	Erythrulose-1-phosphate isomerase	-	2.41
A002374 (EYK1)	CAG77670	Erythrulose kinase	3.77	5.11
A002375 (EYI2)	RDW42622	Erythrulose-4-phosphate isomerase	5.69	7.80
A002376 (EYD1)	RDW42621	Erythritol dehydrogenase	5.02	6.45
A003663 (TKL1)	VBB79039	Transketolase	-3.11	-1.93
A004280 (ZWF1)	RDW30927	Glucose-6-phosphate dehydrogenase	-1.78	-
A005140 (ER10)	RDW31413	Erythrose reductase	-1.36	-
*A005663 (*TAL1*)*	RDW30598	Transaldolase	-1.25	-1.24
A005791 (ER27)	RDW32794	Erythrose reductase	-1.45	-

Table S5 Detailed information of the genes used for overexpression analysis

Gene ID	Protein ID	Product	Log_2FC		
			YLX6 vs YLX3	YLX8 vs YLX3	YLX8 vs YLX6
A000121	SEI35958	YALIA101S09e02762g1_1	9.5	12.0	2.5
A000184	RDW35377	Alpha/Beta hydrolase protein	3.40	4.26	-
A000800	RDW42845	Hypothetical protein B0I74DRAFT_119247	4.3	7.1	2.8
A001678	SEI37011	YALIA101S17e00276g1_1	3.4	5.6	2.2
A002175	RDW32666	Hypothetical protein B0I72DRAFT_164575	4.4	6.2	1.8
A002375	RDW42622	Hypothetical protein B0I73DRAFT_91619	5.7	7.8	2.1
A002808	RDW50623	Hypothetical protein B0I75DRAFT_167065	5.4	8.1	2.7
A003183	SEI35064	YALIA101S06e01156g1_1	2.6	3.5	-
A003902	RDW33865	Hypothetical protein B0I72DRAFT_163816	4.7	7.9	2.6
A004055	RDW34144	Cell wall transcription factor ACE2	2.4	4.8	2.4
A004467	RDW43099	GPR1/FUN34/yaaH family-domain-containing protein	3.0	5.1	2.1
A004535	RDW51119	Hypothetical protein B0I75DRAFT_107230	2.0	7.8	5.8
A004625	RDW34268	Hypothetical protein B0I72DRAFT_19645	4.5	7.4	2.9
A004733	RDW47589	Hypothetical protein B0I74DRAFT_135189	6.6	7.5	-
A005690	RDW31305	GPR1/FUN34/yaaH family-domain-containing protein	4.7	6.8	2.0
A005844	VBB82684	Conserved hypothetical protein	2.0	4.7	2.8
A006220	-	-	3.4	8.2	4.8
A006279	VBB82226	Catalase	2.3	3.8	-

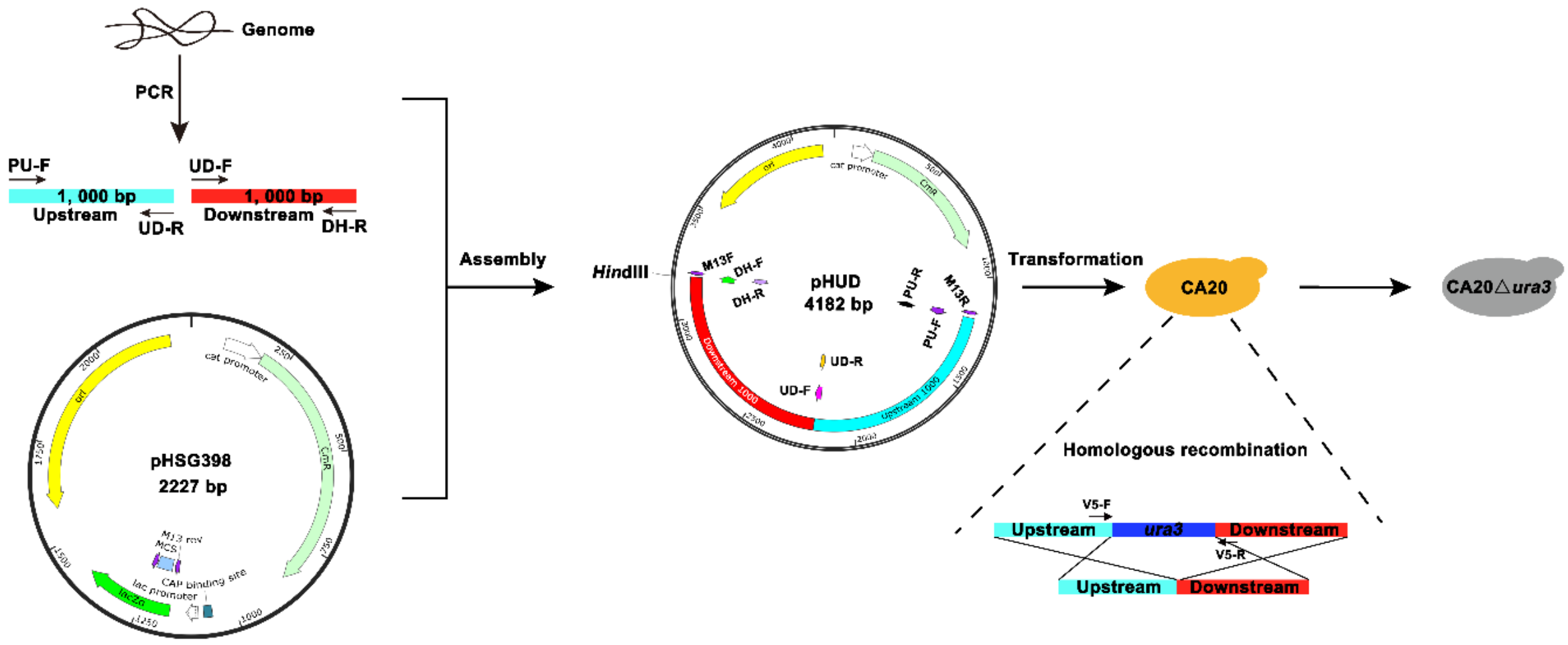

Fig. S1 Scheme flow of the construction of plasmid pHUD. The upstream and downstream DNA fragments of *ura3* (1 kb) were amplified using primers PU-F/UD-R and UD-F/DH-R, respectively. The pHSG398 was linearized by amplification using primers DH-F and PU-R. Then, the upstream DNA fragment, the downstream DNA fragment, and the linearized pHSG398 were assembled using Gibson Assembly Master Mix (New England Biolabs, MA, USA), according to the manufacturer's instructions. The assembled DNA mix was transformed into *E. coli* DH5α to produce the plasmid pHUD, during which the positive recombinant was selected by colony PCR using the primers M13F and M13R. Thereafter, the pHUD was transformed into *Y. lipolytica* CA20 to produce *Y. lipolytica* CA20Δ*ura3*. The mutant strain was verified by sequencing using primers V5-F and V5-R

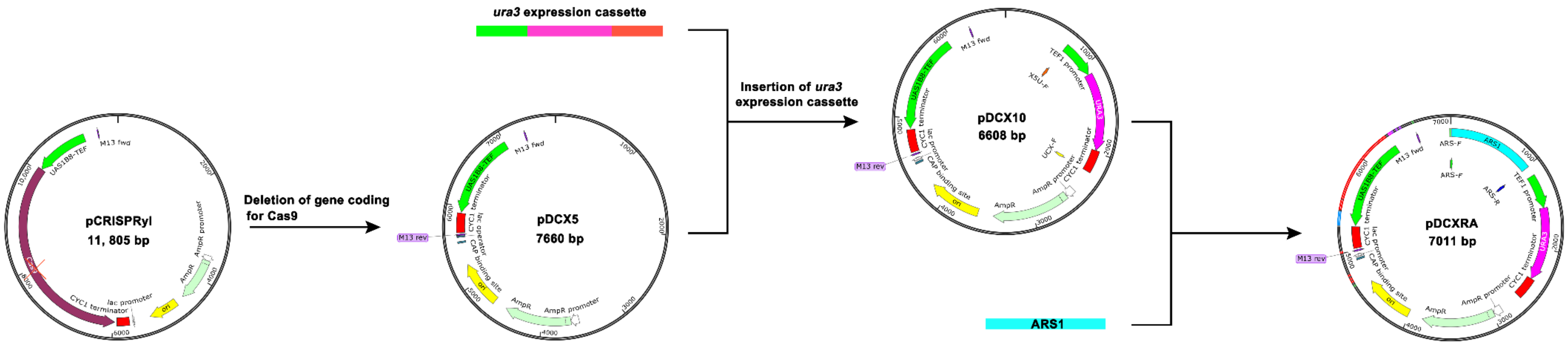

Fig. S2 Scheme flow of the construction of plasmid pDCXRA. The gene coding for Cas9 involved in pCRISPRyl was deleted by PCR amplification using primers CX5-F and CX5-R to produce pDCX5. Then the complete *ura3* expression cassette was synthesized and inserted into the pDCX5 to produce pDCX10 by seamless cloning using Gibson Assembly Master Mix, during which the primers X5U-F/X5U-R and UCX-F/UCX-R were used. Thereafter, the ARS1 fragment was amplified from the CA20 genome using primers ARS-F and ARS-R, and inserted into the plasmid pDCX10 linearized by PCR amplification using primers PARS-F and PARS-R to produce the expression vector pDCXRA by seamless cloning. The pDCXRA was used for the overexpression of genes of interest (*A000121*, *A000184*, *A000800*, *A001678*, *A002175*, *A002375*, *A002808*, *A003183*, *A003902*, *A004055*, *A004467*, *A004535*, *A004625*, *A004733*, *A005690*, *A005844*, *A006220*, and *A006279*). These genes were amplified from the CA20 genome by PCR using primers 121-F/121-R, 184-F/184-R, 800-F/800-R, 1678-F/1678-R, 2175-F/2175-R, 2375-F/2375-R, 2808-F/2808-R, 3183-F/3183-R, 3902-F/3902-R, 4055-F/4055-R, 4467-F/4467-R, 4535-F/4535-R, 4625-F/4625-R, 4733-F/4733-R, 5690-F/5690-R, 5844-F/5844-R, 6220-F/6220-R, and 6279-F/6279-R, respectively. Simultaneously, the plasmid pDCXRA was linearized by using primers P121-F/P121-R, P184-F/P184-R, P800-F/P800-R, P1678-F/P1678-R, P2175-F/P2175-R, P2375-F/P2375-R, P2808-F/P2808-R, P3183-F/P3183-R, P3902-F/P3902-R, P4055-F/P4055-R, P4467-F/P4467-R, P4535-F/P4535-R, P4625-F/P4625-R, P4733-F/P4733-R, P5690-F/P5690-R, P5844-F/P5844-R, P6220-F/P6220-R, and P6279-F/P6279-R, respectively. Then, each of the gene and the linearized pDCXRA were assembled by Gibson Assembly Master Mix to produce the recombinant plasmid

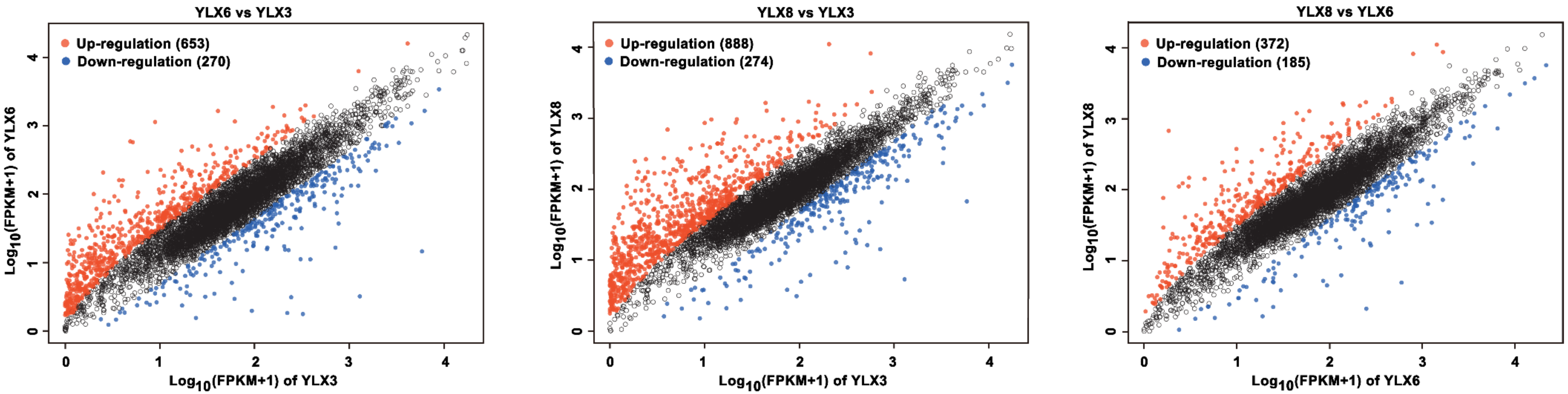

Fig. S3 Visualized distribution of the differentially expressed genes (DEGs) between YLX6 and YLX3, YLX8 and YLX3, and YLX8 and YLX6 by scatter plot

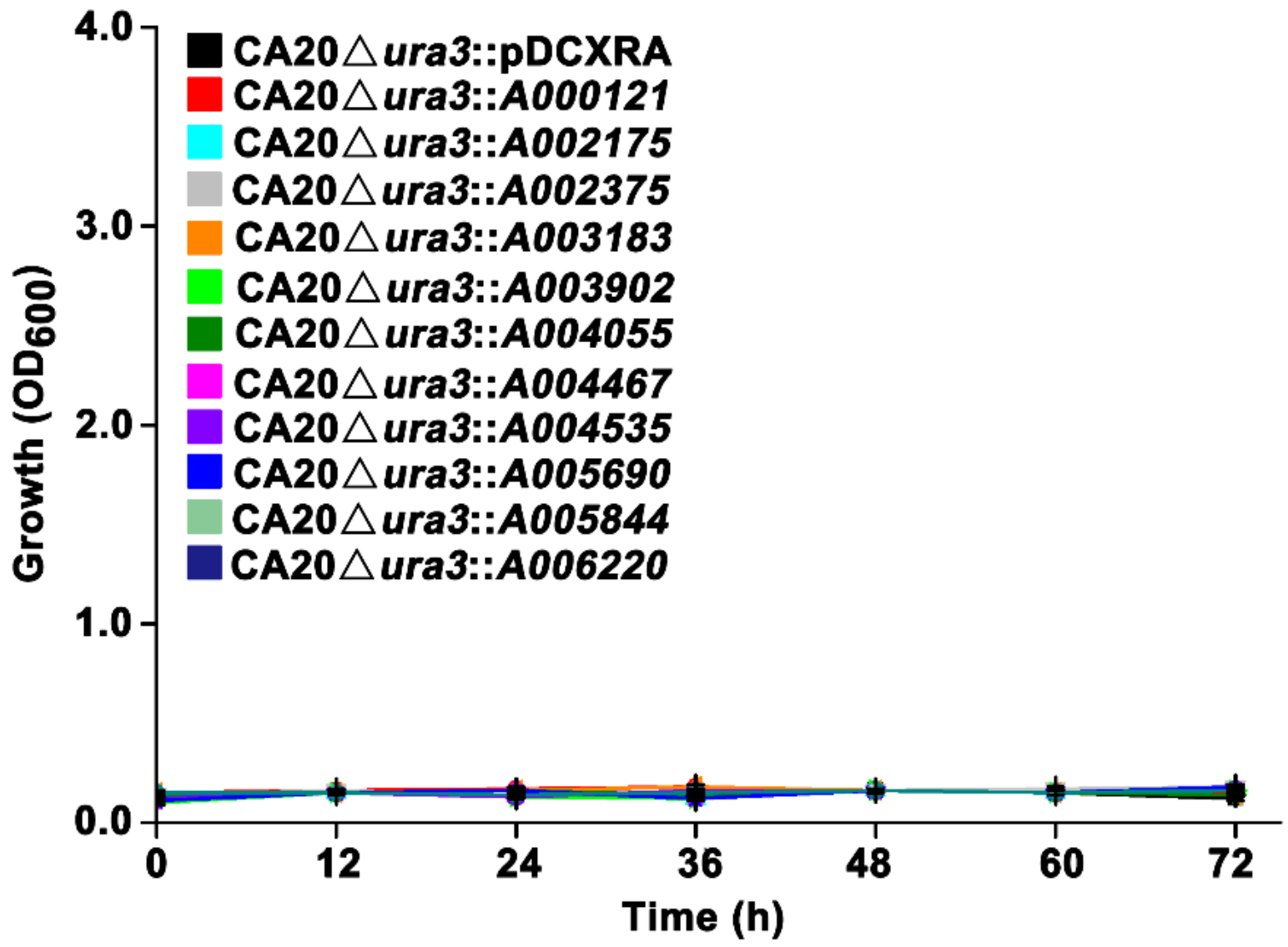

Fig. S4 Growth curves of CA20Δ*ura3* with an overexpression of the upregulated genes at 35 °C. Data obtained from three biological replicates were shown as the mean ± standard deviation.
